# Human vaccination against *Plasmodium vivax* Duffy-binding protein induces strain-transcending antibodies

**DOI:** 10.1172/jci.insight.93683

**Published:** 2017-06-15

**Authors:** Ruth O. Payne, Sarah E. Silk, Sean C. Elias, Kathryn H. Milne, Thomas A. Rawlinson, David Llewellyn, A. Rushdi Shakri, Jing Jin, Geneviève M. Labbé, Nick J. Edwards, Ian D. Poulton, Rachel Roberts, Ryan Farid, Thomas Jørgensen, Daniel G.W. Alanine, Simone C. de Cassan, Matthew K. Higgins, Thomas D. Otto, James S. McCarthy, Willem A. de Jongh, Alfredo Nicosia, Sarah Moyle, Adrian V.S. Hill, Eleanor Berrie, Chetan E. Chitnis, Alison M. Lawrie, Simon J. Draper

**Affiliations:** 1 The Jenner Institute, University of Oxford, Oxford, United Kingdom.; 2 International Center for Genetic Engineering and Biotechnology, Aruna Asaf Ali Marg, New Delhi, India.; 3 QIMR Berghofer Medical Research Institute, Herston, Queensland, Australia.; 4 ExpreS^2^ion Biotechnologies, SCION-DTU Science Park, Hørsholm, Denmark.; 5 Department of Biochemistry, University of Oxford, Oxford, United Kingdom.; 6 Wellcome Trust Sanger Institute, Cambridge, United Kingdom.; 7 ReiThera SRL (formerly Okairòs SRL), Viale Città d’Europa, Rome, Italy.; 8 CEINGE, Naples, Italy.; 9 Department of Molecular Medicine and Medical Biotechnology, University of Naples Federico II, Naples, Italy.; 10 Clinical Biomanufacturing Facility, University of Oxford, Oxford, United Kingdom.; 11 Institut Pasteur, Department of Parasites and Insect Vectors, Paris, France.

**Keywords:** Infectious disease, Vaccines

## Abstract

**BACKGROUND.*** Plasmodium vivax* is the most widespread human malaria geographically; however, no effective vaccine exists. Red blood cell invasion by the *P*. *vivax* merozoite depends on an interaction between the Duffy antigen receptor for chemokines (DARC) and region II of the parasite’s Duffy-binding protein (PvDBP_RII). Naturally acquired binding-inhibitory antibodies against this interaction associate with clinical immunity, but it is unknown whether these responses can be induced by human vaccination.

**METHODS.** Safety and immunogenicity of replication-deficient chimpanzee adenovirus serotype 63 (ChAd63) and modified vaccinia virus Ankara (MVA) viral vectored vaccines targeting PvDBP_RII (Salvador I strain) were assessed in an open-label dose-escalation phase Ia study in 24 healthy UK adults. Vaccines were delivered by the intramuscular route in a ChAd63-MVA heterologous prime-boost regimen using an 8-week interval.

**RESULTS.** Both vaccines were well tolerated and demonstrated a favorable safety profile in malaria-naive adults. PvDBP_RII–specific ex-vivo IFN-γ T cell, antibody-secreting cell, memory B cell, and serum IgG responses were observed after the MVA boost immunization. Vaccine-induced antibodies inhibited the binding of vaccine homologous and heterologous variants of recombinant PvDBP_RII to the DARC receptor, with median 50% binding-inhibition titers greater than 1:100.

**CONCLUSION.** We have demonstrated for the first time to our knowledge that strain-transcending antibodies can be induced against the PvDBP_RII antigen by vaccination in humans. These vaccine candidates warrant further clinical evaluation of efficacy against the blood-stage *P*. *vivax* parasite.

**TRIAL REGISTRATION.** Clinicaltrials.gov NCT01816113.

**FUNDING.** Support was provided by the UK Medical Research Council, UK National Institute of Health Research Oxford Biomedical Research Centre, and the Wellcome Trust.

## Abstract 2

A clinical trial of a candidate blood-stage Plasmodium vivax vaccine targeting the Duffy-binding protein demonstrates safety and immunogenicity in healthy adults and induces strain-transcending antibodies.

## Introduction

Five species of *Plasmodium* parasite are known to cause malaria following human infection, with *P*. *falciparum* the major causative agent of deaths in sub-Saharan Africa and thus historically the dominant focus of vaccine development efforts (1). However, a second parasite species, *P*. *vivax*, is more widespread geographically and also constitutes a significant proportion of human malaria cases. Indeed, recent data suggest 2.5 billion people are living at risk of *P*. *vivax* infection in the Americas, Central and Southeast Asia (2), as well as Africa (3), highlighting significant levels of morbidity that have been chronically underappreciated (4). Consequently, the revised Malaria Vaccine Technology Roadmap to 2030 (5) now recognizes the importance of *P*. *vivax* and calls for a vaccine to achieve 75% efficacy over 2 years — equally weighted with *P*. *falciparum* in an era of renewed political will to move towards malaria elimination and eradication.

Different vaccine strategies target different stages of the malaria parasite’s complex life cycle. To date, 2 subunit vaccines targeting the pre-erythrocytic stage *P*. *vivax* circumsporozoite protein (PvCSP), based on recombinant protein- or long synthetic peptide–in-adjuvant formulations, have reached clinical trials (6, 7). The soluble recombinant protein candidate, VMP001, delivered in GlaxoSmithKline’s (GSK’s) proprietary Adjuvant System AS01B, showed robust immunogenicity in healthy US volunteers but failed to induce sterile protection following *P*. *vivax* controlled human malaria infection (CHMI) using a mosquito bite protocol; however, a small but significant delay in time to parasitemia was seen in 16 of 27 vaccinated subjects compared with the control group (7). A virus-like particle (VLP) using the same antigen fused to hepatitis B surface antigen (HBsAg), expressed in *Saccharomyces cerevisiae* and designated CSV-S,S, showed modest improvements in immunogenicity when tested in rhesus macaques with AS01 (8), but has not progressed to clinical testing. In 2 other phase Ia clinical trials, a soluble recombinant protein vaccine targeting the sexual-stage ookinete surface protein Pvs25 was tested in 2 different adjuvants. This vaccine candidate, called Pvs25H, showed transmission-blocking activity in a direct membrane feeding assay when formulated with Alhydrogel (9), but vaccinations with Montanide ISA 51 were halted due to unexpected reactogenicity (10). None of these pre-erythrocytic or transmission-blocking candidate subunit vaccines remain in active clinical development.

Vaccines targeting the asexual blood-stage infection form an alternative and complementary approach to vaccines against the other life cycle stages, seeking to control and clear parasitemia in order to prevent clinical disease and death as well as onward transmission. Although many candidates have been assessed over the years for *P*. *falciparum* (1), no clinical trials of vaccines against merozoite ligands involved in erythrocyte invasion have been reported for *P*. *vivax* (11). The Duffy-binding proteins (DBPs), or erythrocyte-binding ligands/antigens (EBL/EBA), are a family of micronemal parasite proteins that are functionally conserved across *Plasmodium* species. All parasites have at least one EBL, and in many cases these lead to redundancy, as has been well established in *P*. *falciparum* (12). However in the case of *P*. *vivax*, invasion of host red blood cells (RBC) is restricted to CD71^+^ reticulocytes (13) and believed to necessitate the interaction of the *P*. *vivax* Duffy-binding protein (PvDBP) with the human Duffy antigen receptor for chemokines (DARC/Fy) (14). Notably, Duffy-negative individuals are protected from blood-stage *P*. *vivax* infection, an observation first reported by Miller et al. in 1976 (15), confirmed by CHMI studies (16), and associated geographically with low-level endemicity in sub-Saharan Africa (3). Consistent with this, genetic knockout of the orthologous simian malaria *P*. *knowlesi* DBPα gene also prevents invasion of Duffy-positive erythrocytes in vitro (17). However, this paradigm of an essential RBC invasion pathway has been challenged in recent years with reports of *P*. *vivax* infection in Duffy-negative individuals (3, 18) and a growing appreciation of the complexity of other families of invasion ligands, such as the reticulocyte-binding proteins (PvRBPs) (19). In parallel, a PvDBP gene duplication in *P*. *vivax* isolates (20) has also been reported, likely representing a second erythrocyte-binding protein (EBP2) (21), although studies have not linked this gene to Duffy-negative infection (22, 23). Therefore, although the complete molecular basis of *P*. *vivax* invasion into DARC-negative erythrocytes remains unknown, it may still involve PvDBP.

In the case of PvDBP, a conserved, extracellular, cysteine-rich region known as region II (PvDBP_RII) contains the receptor-binding domain of PvDBP. Structural analyses of this domain have shown that PvDBP_RII dimers bind either 1 or 2 DARC ectodomains, creating distinct heterotrimeric and heterotetrameric architectures (24, 25). Immunization of mice, rabbits, and nonhuman primates (NHPs) using PvDBP_RII–based vaccines induces binding-inhibitory antibodies (BIAbs) (26–28), and those raised against the *P*. *knowlesi* DBPα ortholog can block RBC invasion by this parasite in vitro (29). In humans, naturally acquired high-titer BIAbs against PvDBP_RII have been associated with reduced risk of *P*. *vivax* infection, lower *P*. *vivax* parasite densities following infection, and decreased risk of clinical malaria (30, 31). Consequently, PvDBP_RII remains the most promising subunit vaccine target against *P*. *vivax* merozoites; however, this antigen has never progressed to clinical trials and no data are available on the ability of vaccines to induce effective immune responses in humans.

With regard to antibody induction by vaccination, the mainstay approach has been the development of recombinant protein- or VLP-in-adjuvant formulations. An alternative strategy has used recombinant viral vectored vaccines to deliver protein antigens of interest with the key aim of inducing antibodies in conjunction with T cell responses. The most successful approach to date has utilized a recombinant replication-deficient adenovirus (of human or simian serotype) to prime the immune response, followed by a booster vaccination (typically 8 weeks later) with an attenuated poxvirus recombinant for the same antigen (32). These vectors have shown high-titer antibody induction against numerous difficult-to-express malaria antigens in animal models, including NHPs (33, 34). We, and others, have previously reported such viral vectored vaccines to be safe and immunogenic for T cells and antibodies in healthy adult UK and US volunteers when delivering numerous *P*. *falciparum* antigens, including the pre-erythrocytic antigen multiple-epitope string fused to thrombospondin-related adhesion protein (ME-TRAP) (35) and circumsporozoite protein (PfCSP) (36), as well as the blood-stage antigens merozoite surface protein 1 (PfMSP1) (37) and apical membrane antigen 1 (PfAMA1) (38, 39). In 2014 and 2015, the same adenovirus-poxvirus vectored vaccine technologies were developed rapidly for Ebola (40).

Here, we report the safety and immunogenicity of a similar approach in an open-label dose-escalation phase Ia study in healthy UK adults using replication-deficient chimpanzee adenovirus serotype 63 (ChAd63) and the attenuated orthopoxvirus modified vaccinia virus Ankara (MVA) encoding PvDBP_RII from the Salvador I (SalI) reference strain of *P*. *vivax*. These vaccines have been previously shown to be immunogenic in mice and rabbits (27). Now we show that these vaccines demonstrate a favorable safety profile in malaria-naive adults, and confirm to our knowledge for the first time that substantial PvDBP_RII–specific antibodies and B cell and T cell responses can be induced by immunization in humans. Vaccine-induced serum antibodies were capable of inhibiting the in vitro binding of vaccine homologous and heterologous variants of recombinant PvDBP_RII to the DARC receptor.

## Results

### Twenty-four healthy adult volunteers were enrolled into the VAC051 trial to test the ChAd63-MVA PvDBP_RII vaccine in an open-label, dose-escalation study design.

**Figure 1:**
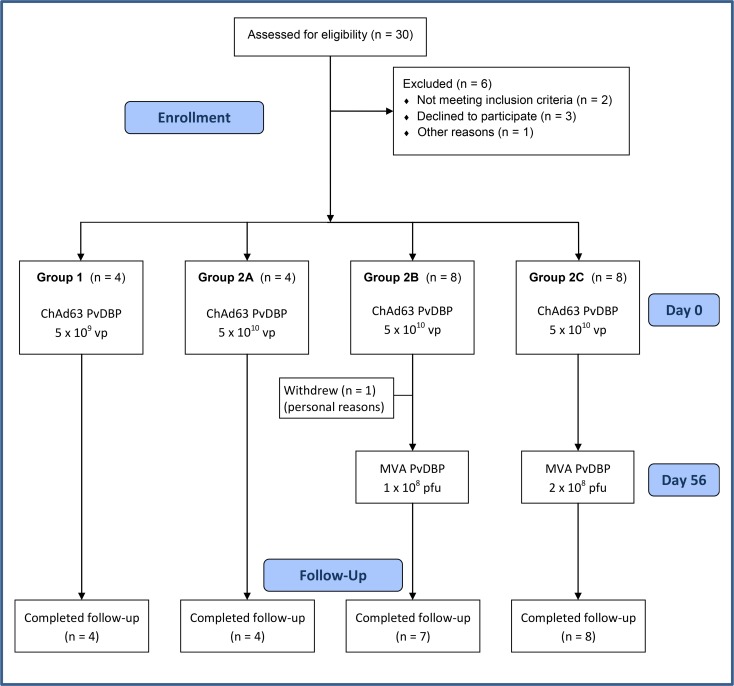
VAC051 flow chart of study design and volunteer recruitment. Recruitment for the VAC051 study took place between May 2013 and February 2014. The final follow-up visit took place in July 2014. All immunizations were administered intramuscularly, with sequential vaccines administered into the deltoid of alternating arms.

Thirty UK adult volunteers were screened in total, of which 24 were enrolled (Figure 1). Four volunteers were recruited to groups 1 and 2A, and 8 volunteers to groups 2B and 2C. In total, 15 females and 9 males were enrolled. The mean age of volunteers was 25 years 9 months (range 18–40 years). Four volunteers were enrolled into group 1 and received 5 × 10^9^ viral particles (vp) of the ChAd63 PvDBP_RII vaccine. Following a safety review, the dose of ChAd63 PvDBP_RII was increased to 5 × 10^10^ vp for group 2. Four volunteers in group 2A received ChAd63 PvDBP_RII alone, while volunteers in groups 2B and 2C received ChAd63 PvDBP_RII followed 8 weeks later with a boost vaccination of MVA PvDBP_RII at a dose of 1 × 10^8^ plaque-forming units (PFU) or 2 × 10^8^ PFU, respectively. One volunteer withdrew from group 2B prior to the MVA PvDBP_RII vaccination due to personal commitments and was not replaced, resulting in 23 volunteers completing follow-up as per protocol.

### ChAd63 and MVA PvDBP_RII show a favorable safety profile in healthy UK adult volunteers.

**Figure 2:**
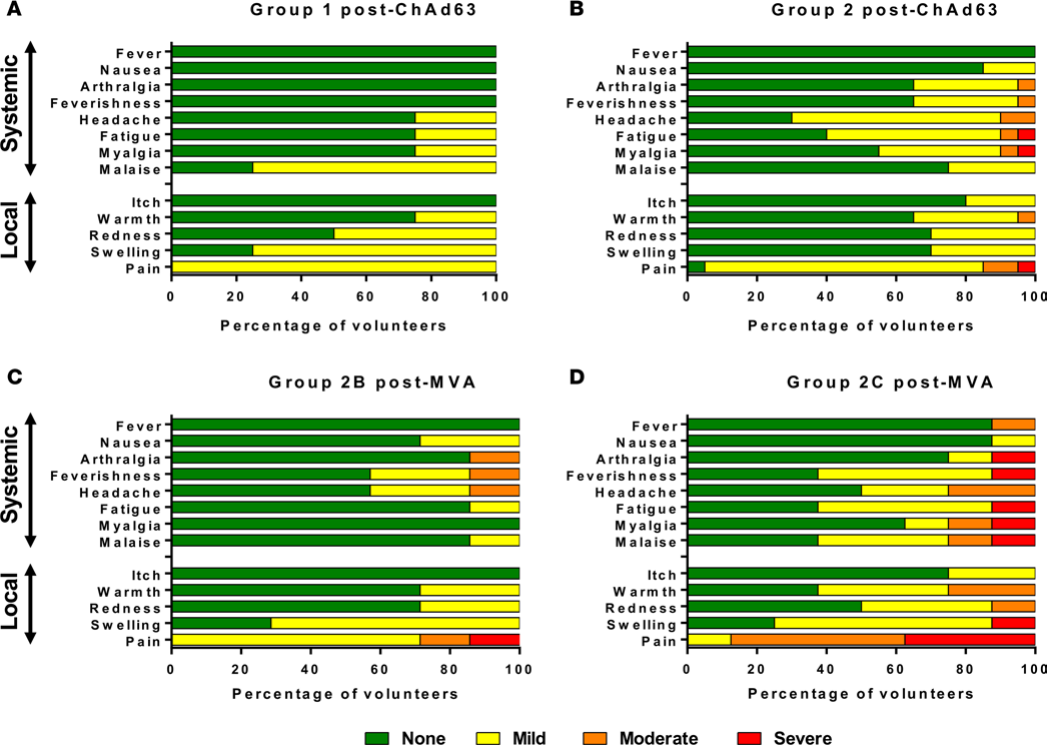
Solicited AEs following vaccination with ChAd63 and MVA PvDBP_RII. The solicited local and systemic adverse events (AEs) recorded for 14 days following ChAd63 PvDBP_RII and for 7 days following MVA PvDBP_RII are shown at the maximum severity reported by all volunteers. (**A**) Four volunteers received 5 × 10^9^ viral particles (vp) ChAd63 PvDBP_RII (group 1), and (**B**) 20 received 5 × 10^10^ vp (group 2). (**C**) Seven of the group 2 volunteers went on to receive MVA PvDBP_RII 1 × 10^8^ PFU (group 2B), and (**D**) 8 received 2 × 10^8^ PFU (group 2C). ChAd63, replication-deficient chimpanzee adenovirus serotype 63; MVA, modified vaccinia virus Ankara; PvDBP_RII, region II of the *P*. *vivax* Duffy-binding protein.

There were no serious adverse events (AEs) or unexpected reactions during the course of the trial and no volunteers withdrew due to vaccine-related AEs. ChAd63 PvDBP_RII and MVA PvDBP_RII demonstrated favorable safety profiles, similar to those seen in previous clinical trials with the same viral vectors recombinant for *P*. *falciparum* malaria antigens (35–38). All AEs following ChAd63 PvDBP_RII 5 × 10^9^ vp were mild, as were the vast majority in group 2, although some volunteers did report moderate or severe AEs following immunization with the full dose. The higher dose of MVA PvDBP_RII was more reactogenic than the lower dose, with half of the volunteers reporting at least 1 severe AE, although no systemic AE was reported as severe for more than 24 hours. The maximum severities of solicited local and systemic AEs reported by volunteers following each vaccination are shown in Figure 2. All unsolicited AEs considered possibly, probably, or definitely related to either vaccination were mild in nature (Supplemental Table 1; supplemental material available online with this article; https://doi.org/10.1172/jci.insight.93683DS1). There was only 1 laboratory AE following ChAd63 PvDBP_RII that was considered possibly, probably, or definitely related to vaccination: a mild lymphopenia in 1 volunteer vaccinated with 5 × 10^10^ vp. Similarly, there was only 1 laboratory AE following MVA PvDBP_RII that was considered possibly, probably, or definitely related to vaccination: a moderate eosinophilia in 1 volunteer vaccinated with 1 × 10^8^ PFU, which peaked more than 4 weeks after vaccination. Both of these laboratory AEs resolved spontaneously.

### ChAd63 and MVA PvDBP_RII expand IFN-γ T cell responses in healthy UK adult volunteers.

**Figure 3:**
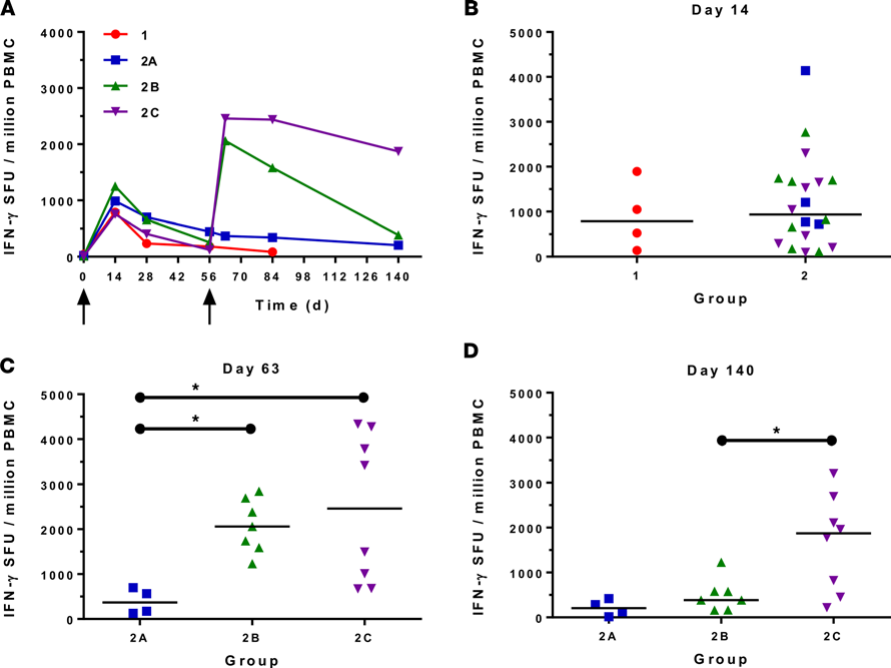
Ex-vivo IFN-γ T cell response to vaccination. (**A**) Median ex vivo IFN-γ ELISPOT responses in peripheral blood mononuclear cells (PBMCs) to the PvDBP_RII insert (summed response across all the individual peptide pools) shown for all groups. Individual responses are shown in Supplemental Figure 1. Median and individual responses are shown at (**B**) day 14, (**C**) day 63, and (**D**) day 140. Symbols are coded according to group. *P < 0.05. Responses between groups 1 (*n* = 4) and 2 (*n* = 20) at day 14, and between groups 2B (*n* = 7) and 2C (*n* = 8) at day 140 were assessed by Mann-Whitney test (**B** and **D**); responses between groups 2A (*n* = 4), 2B (*n* = 7), and 2C (*n* = 8) at day 63 were assessed by Kruskal-Wallis test with Dunn’s multiple comparison test (**C**). SFU, spot-forming units; PvDBP_RII, region II of the *P*. *vivax* Duffy-binding protein.

The kinetics and magnitude of the PvDBP-specific T cell response were assessed over time by ex vivo IFN-γ ELISPOT following restimulation of peripheral blood mononuclear cells (PBMCs) with 20-mer peptides overlapping by 10 amino acids (aa) spanning the entire PvDBP_RII insert present in the vaccines (Figure 3). Vaccination with ChAd63-MVA PvDBP_RII induced antigen-specific T cell responses in all volunteers, with individual responses shown in Supplemental Figure 1 and median responses to the total vaccine insert shown for each group in Figure 3A. Following ChAd63 PvDBP_RII prime, there was no significant difference between median responses in the lower-dose group 1 in comparison with group 2 at the peak of the response on day 14 (median 787 [range 140–1,893] vs. 937 [range 96–4,141] spot-forming units [SFU]/million PBMCs in groups 1 versus 2, respectively; *n* = 4 vs. 20, *P* = 0.79 by Mann-Whitney test) (Figure 3B). Responses subsequently followed classical T cell kinetics and contracted by day 56 (Figure 3A). Administration of MVA PvDBP_RII significantly boosted these responses in all volunteers as measured 1 week later on day 63 (groups 2B and 2C vs. 2A, Kruskal-Wallis test with Dunn’s multiple comparison test) (Figure 3C), reaching medians of 2,061 (range 1,232–2,844) and 2,459 (range 675–4,336) SFU/million PBMCs in groups 2B and 2C, respectively, versus 368 (range 127–699) SFU/million PBMCs in group 2A. However, there was no significant difference between the 2 groups who received the different doses of MVA PvDBP_RII (*P* = 0.96, Mann-Whitney test). T cell responses were spread across the whole PvDBP_RII antigen, with responses detected in all 6 of the peptide pools used in the ELISPOT assay (Supplemental Figure 2). Following the peak at day 63, responses contracted but were maintained above baseline at the end of the study period, with significantly better maintained responses at day 140 in group 2C as compared with group 2B (median 1,871 vs. 385 SFU/million PBMCs) (*P* = 0.03, Mann-Whitney test) (Figure 3D).

### ChAd63 and MVA PvDBP_RII induce serum antibody responses and memory B cells in healthy UK adult volunteers.

**Figure 4:**
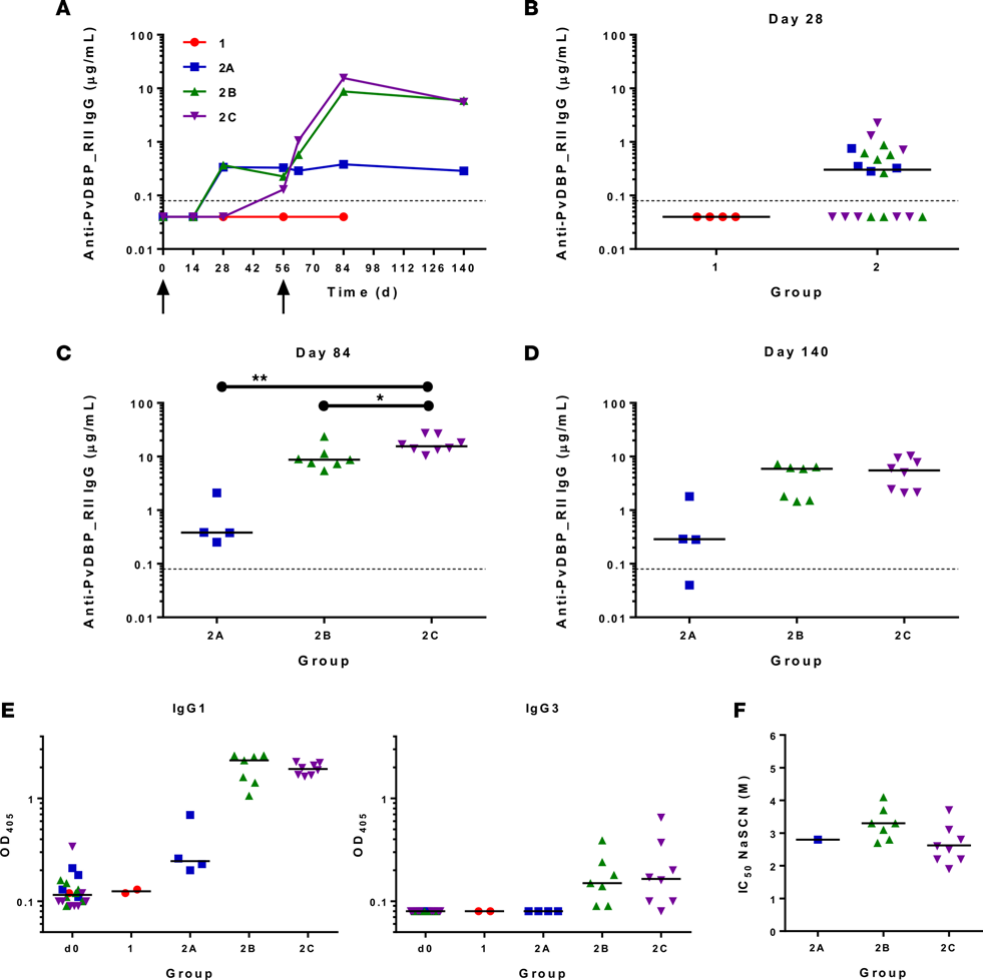
Serum antibody response to vaccination. (**A**) Median anti–PvDBP_RII serum total IgG responses shown for all groups over time. Individual responses are shown in Supplemental Figure 3. Median and individual responses are shown at (**B**) day 28, (**C**) day 84, and (**D**) day 140. The horizontal dotted line indicates the limit of detection of the assay. (**E**) Isotype profiles of serum antibody responses were assessed by ELISA. Responses are shown at baseline (d0) and for all groups at day 84. Individual and median responses are shown for IgG1 and IgG3; results for IgG2, IgG4, IgA, and IgM are shown in Supplemental Figure 6. (**F**) Avidity of serum IgG responses at day 84 was assessed by NaSCN-displacement PvDBP_RII ELISA and is reported as the molar (M) concentration of NaSCN required to reduce the starting OD in the ELISA by 50% (IC_50_). Symbols are coded according to group. *P < 0.05, **P < 0.01. Responses in groups 2A (*n* = 4), 2B (*n* = 7), and 2C (*n* = 8) were assessed by Kruskal-Wallis test with Dunn’s multiple comparison test; responses between groups 2B and 2C were assessed by Mann-Whitney test (**C**). PvDBP_RII, region II of the *P*. *vivax* Duffy-binding protein.

The kinetics and magnitude of the anti–PvDBP_RII serum IgG antibody response were assessed over time by ELISA against recombinant protein (Figure 4). Priming vaccination with 5 × 10^10^ vp ChAd63 PvDBP_RII followed by MVA PvDBP_RII boost induced antigen-specific IgG responses in all volunteers (groups 2B and 2C), with individual responses shown in Supplemental Figure 3 and median responses shown for each group in Figure 4A. Responses are reported in μg/ml following conversion of ELISA arbitrary units (AU) by calibration-free concentration analysis (CFCA) (Supplemental Figure 4). Following ChAd63 PvDBP_RII prime with 5 × 10^9^ vp, none of the 4 volunteers showed a detectable response on day 28, in contrast to 12 of 20 volunteers who did show a response (median 0.3, range 0–2.3 μg/ml, *n* = 20) following priming with 5 × 10^10^ vp (*P* = 0.07, Mann-Whitney test) (Figure 4B). Responses were subsequently maintained in group 2 volunteers prior to administration of MVA PvDBP_RII, which led to a boost as measured 4 weeks later on day 84 (Figure 4A) — this reached significance for group 2C versus 2A (Kruskal-Wallis test with Dunn’s multiple comparison test) (Figure 4C). Responses in group 2C (median 15.6, range 10.5–27.2 μg/ml, *n* = 8) were also modestly, but significantly, higher than in group 2B (median 8.8, range 5.5–23.7 μg/ml, *n* = 8) at this peak time point (*P* = 0.014, Mann-Whitney test). Serum antibody responses decreased by day 140 but were well maintained above preboost levels, with no significant difference between groups 2B and 2C (*P* = 0.34, Mann-Whitney test) (Figure 4D). Day 84 plasma were also tested against a panel of overlapping 20-mer linear peptides; however, few responses were detected above background, suggesting the vast majority of vaccine-induced anti–PvDBP_RII IgG recognize conformational, as opposed to linear, epitopes (Supplemental Figure 5).

The serum antibody response against PvDBP_RII as measured by ELISA at day 84 was composed of IgG1 and modest levels of IgG3 (Figure 4E), with little to no IgG2, IgG4, IgA, or IgM detectable above baseline (day 0) levels (Supplemental Figure 6). The avidity of the anti–PvDBP_RII IgG, as measured by a NaSCN-displacement ELISA, was similar at day 84 for all volunteers in groups 2B and 2C, with the IC_50_ ranging from 1.9 to 4.1 M. Avidity could only be measured for 1 vaccinee in group 2A at this time point with an IC_50_ of 2.8 M, suggesting no change following MVA PvDBP_RII boost (Figure 4F).

**Figure 5:**
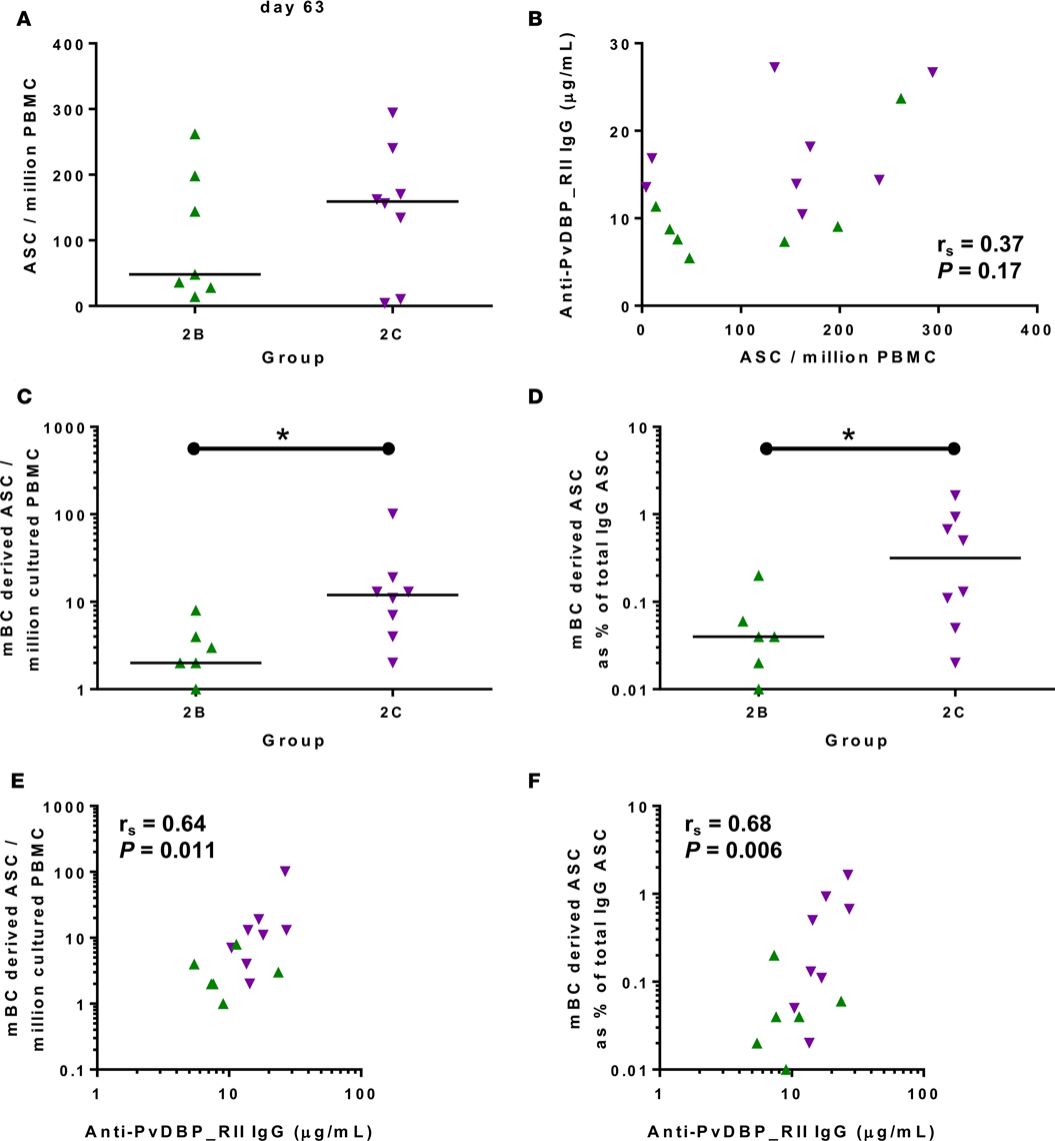
B cell response to vaccination. (**A**) PvDBP_RII–specific antibody-secreting cell (ASC) responses were assessed by ex-vivo ELISPOT using PvDBP_RII protein and frozen peripheral blood mononuclear cells (PBMCs) from the day 63 time point. Individual and median responses are shown for each group and reported as PvDBP_RII–specific ASCs per million PBMCs used in the assay. (**B**) Correlation of the ASC response versus the concentration of serum anti–PvDBP_RII IgG measured at day 84. (**C**) PvDBP_RII–specific memory B cell (mBC) responses were assessed by ELISPOT assay using PvDBP_RII protein. Frozen PBMCs were thawed and underwent a 6-day polyclonal restimulation during which ASCs were derived from mBCs, before testing in the assay. Individual and median responses are shown from the day 84 time point and are reported as mBC-derived PvDBP_RII–specific ASCs per million cultured PBMCs or as (**D**) percentage of total number of IgG-secreting ASCs. (**E** and **F**) Correlations of the mBC response versus the concentration of serum anti–PvDBP_RII IgG at day 84. For all correlations, Spearman’s rank correlation coefficient (*r*_s_) and *P* value are shown. *P < 0.05. Responses between groups 2B (*n* = 7) and 2C (*n* = 8) were assessed by Mann-Whitney test. PvDBP_RII, region II of the *P*. *vivax* Duffy-binding protein.

Previous studies have shown that antibody-secreting cells (ASCs) can be detected in peripheral blood for a short time (around day 7) after MVA boost when using the ChAd63-MVA regimen (41, 42). PvDBP_RII–specific ASC responses were assessed by ex-vivo ELISPOT using frozen PBMCs collected at the day 63 visit for volunteers in groups 2B and 2C. Median responses of 49 versus 159 ASCs per million PBMCs were observed, respectively, but there was no significant difference between the 2 groups (*P* = 0.69, Mann-Whitney test) (Figure 5A). ASC responses across both groups showed a trend to associate with peak serum antibody responses at day 84, but this did not reach significance (Figure 5B).

Memory B cell (mBC) responses were also measured using an established cultured ELISPOT protocol, whereby mBCs within PBMCs undergo a 6-day polyclonal stimulation to form ASCs, which are then measured using the same protocol as for the ex vivo assay. These were measured for volunteers in groups 2B and 2C at the day 84 time point (4 weeks after MVA boost) — most consistently identified as the peak of the mBC response in other trials of ChAd63-MVA *P*. *falciparum* blood-stage malaria vaccines (41, 42). Responses are reported as number of mBC-derived PvDBP_RII–specific ASCs per million cultured PBMCs (Figure 5C), and as a percentage of total IgG-secreting ASCs (Figure 5D); in both cases these were significantly higher in group 2C than 2B (Mann-Whitney test). These mBC responses across both groups also significantly correlated with peak serum antibody responses at day 84 (Figure 5, E and F).

### Vaccine-induced antibodies inhibit PvDBP_RII-DARC binding in vitro.

**Figure 6:**
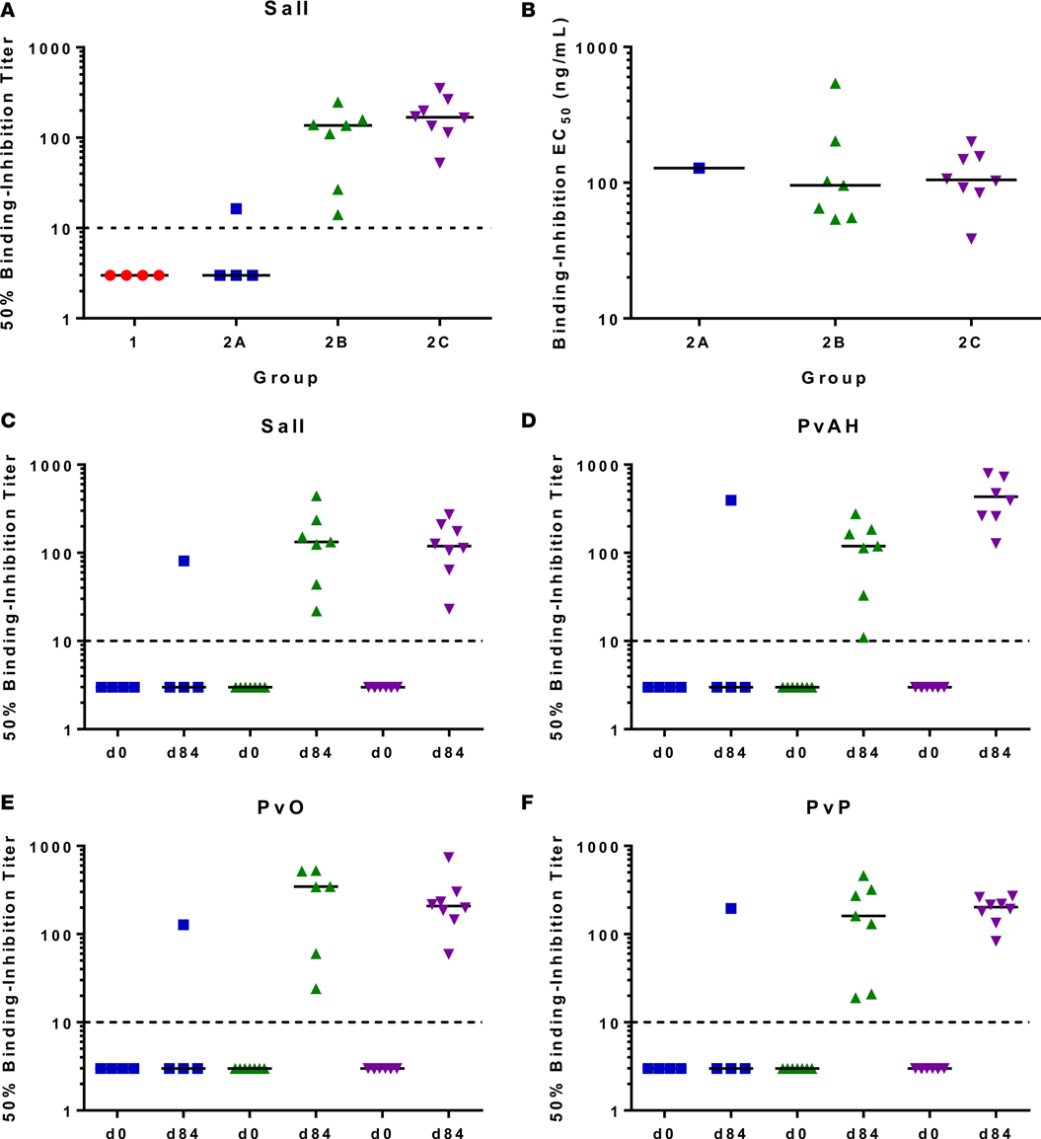
PvDBP_RII–DARC in vitro binding inhibition. (**A**) Day 84 sera from volunteers in groups 1 (*n* = 4), 2A (*n* = 4), 2B (*n* = 7), and 2C (*n* = 8) were tested for their ability to inhibit binding of recombinant PvDBP_RII (SalI) to the Duffy antigen receptor for chemokines (DARC) using an ELISA-based assay in Oxford. Samples were titrated starting at 1:5 dilution down to 1:640 (Supplemental Figure 7A). Data show the interpolated dilution for each sample that gave 50% binding inhibition. (**B**) For positive samples in **A** (*n* = 16), the concentration of anti–PvDBP_RII (SalI) serum IgG that gives 50% binding inhibition (EC_50_) was calculated by dividing the serum ELISA μg/ml by the 50% binding-inhibition serum titer. The result is reported in ng/ml. (**C**–**F**) Day 0 and day 84 sera were assessed as in **A** using the assay established at ICGEB, India, using 4 recombinant alleles of PvDBP_RII: SalI, PvAH, PvO, and PvP. In all panels, the individual and median results are shown for each group. The dashed line shows an arbitrary cutoff below which negative samples are plotted. PvDBP_RII, region II of the *P*. *vivax* Duffy-binding protein; SalI, Salvador I reference strain.

We next assessed the ability of vaccine-induced serum IgG to inhibit binding of recombinant vaccine-homologous PvDBP_RII (SalI) to its receptor (in this case the recombinant N-terminal region of DARC), using an in vitro ELISA methodology in Oxford. Day 84 sera were tested using a 2-fold dilution series starting at 1:5 and through to 1:640, with percentage binding inhibition calculated for each volunteer using their matched day 0 serum sample as the baseline control. Example binding-inhibition curves are shown (Supplemental Figure 7A), and 50% binding-inhibition titers were interpolated from these data (Figure 6A). One sample in group 2A showed a weak 50% binding-inhibition titer of 1:16. All samples from groups 2B and 2C showed binding inhibition with median 50% titers of 1:137 (range 1:14–1:248) and 1:168 (range 1:52–1:352), respectively. To further assess the quality of the vaccine-induced antibody response, these titers were used to calculate the concentration of anti–PvDBP_RII polyclonal IgG that gives 50% binding inhibition in each individual (Figure 6B). Across all groups, the median levels were comparable, requiring 128, 96, and 105 ng/ml in groups 2A, 2B, and 2C, respectively. However, there was over a 10-fold range across all 16 individuals, with the best responder (in group 2C) only requiring 39 ng/ml, versus the worst responder requiring 540 ng/ml (in group 2B). These data suggest that interindividual qualitative differences exist in terms of the binding-inhibitory capacity of the polyclonal vaccine–induced IgG response.

Given that naturally acquired binding-inhibitory anti–PvDBP_RII antibodies can be strain specific (43, 44), we next proceeded to test the day 0 and 84 sera from group 2 against an established panel of recombinant PvDBP_RII alleles (Table 1) using methodology developed at ICGEB, India (Figure 6, C–F). No binding inhibition was observed for any of the day 0 samples against any PvDBP_RII variant. Data for the SalI variant showed very similar results to those observed with the Oxford assay (Spearman’s correlation *r*_s_ = 0.67, *P* = 0.002, *n* = 19). Day 84 sera also showed similar 50% binding-inhibition profiles for the 3 other variants of PvDBP_RII (PvAH, PvO, and PvP), with the same sample positive in group 2A, and median 50% binding-inhibition titers greater than 1:100 for both groups 2B and 2C for all test variants. At the individual level, all samples showed binding inhibition against each variant of PvDBP_RII, but the 50% binding-inhibition titers were variable, again consistent with qualitative differences in each polyclonal response (Supplemental Figure 7B). Interestingly, the individual titers were frequently highest against the vaccine-heterologous PvAH or PvO alleles.

**Figure 7:**
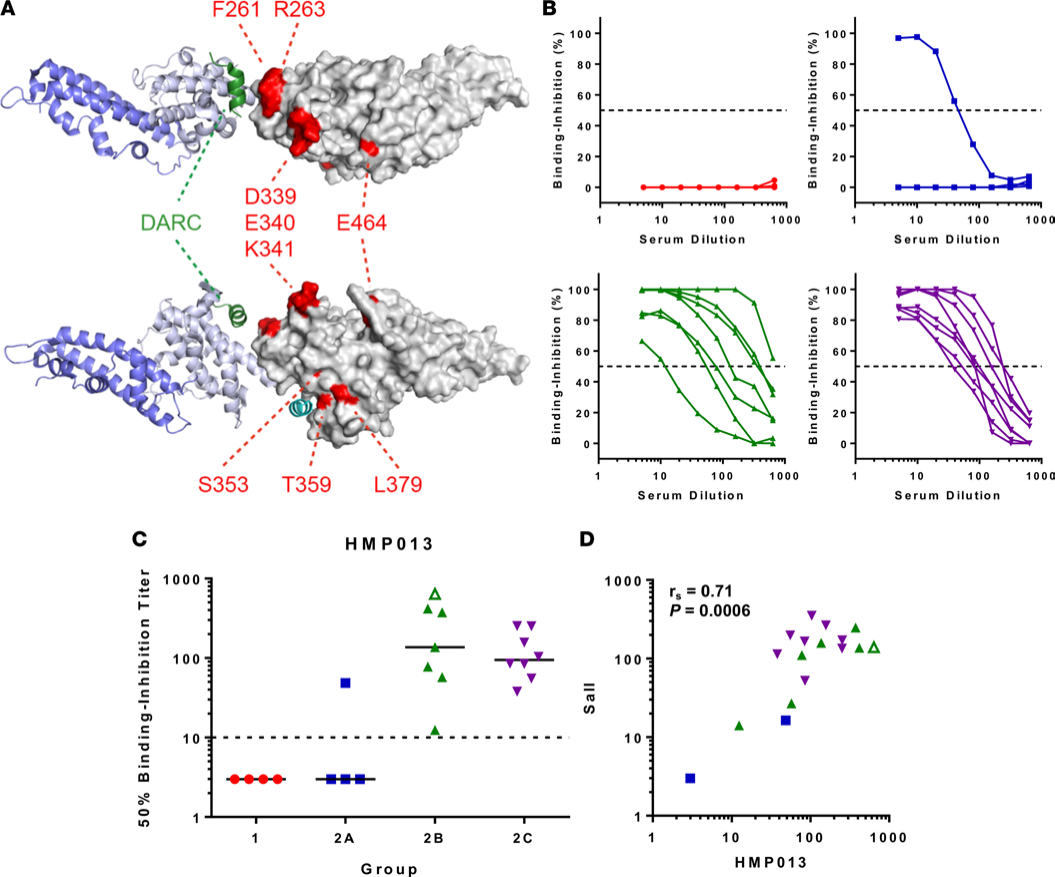
Binding inhibition of the P. vivax HMP013 strain DBP_RII. (**A**) The location of polymorphic residues in PvDBP_RII (HMP013 strain) have been marked on a structure of the PvDBP_RII (SalI strain) dimer bound to the Duffy antigen receptor for chemokines (DARC) aa 19–30 (PDB code 4NVU) (24). Two views of the dimer are shown, rotated by 90 degrees around the horizontal axis. One molecule of PvDBP_RII is shown in gray surface representation with polymorphic residues colored in red. The second molecule of PvDBP_RII is in blue cartoon representation with SD3 in a darker blue. The 2 helices from DARC are shown in green and cyan, respectively. (**B**) Day 84 sera from volunteers in groups 1 (*n* = 4), 2A (*n* = 4), 2B (*n* = 7), and 2C (*n* = 8) were tested for their ability to inhibit binding of recombinant PvDBP_RII (HMP013) to DARC using the ELISA-based assay in Oxford. Samples were titrated starting at 1:5 dilution down to 1:640. Dashed line indicates 50% binding inhibition. Groups coded by color and symbol. (**C**) Data show the interpolated dilution for each sample that gave 50% binding inhibition. One sample in group 2B did not reach 50% binding inhibition by 1:640 dilution and is plotted at this final titer with open triangle symbol. (**D**) Correlation of 50% binding-inhibition titers for the SalI and HMP013 alleles of PvDBP_RII measured using the assay in Oxford. Spearman’s rank correlation coefficient (*r*_s_) and *P* value are shown (*n* = 19). PvDBP_RII, region II of the *P*. *vivax* Duffy-binding protein.

Finally we tested the day 84 sera against an allele of PvDBP_RII present in the HMP013 Indian strain of *P*. *vivax*, which has recently been cryobanked for use as an inoculum in blood-stage CHMI clinical trials (16). After generating a draft assembly of HMP013 (see supplementary material), analysis of the PvDBP_RII sequence from this strain (Table 1) revealed 10 polymorphic positions, of which 5 were not shared with the other variants tested in this study, including some in subdomain 2 (SD2) close to the site shown to bind to aa 19–30 of DARC (Figure 7A). Recombinant PvDBP_RII (HMP013) was subsequently generated and used in the Oxford assay. Binding-inhibition curves were similar to those previously observed with the SalI allele (Figure 7B). Fifty percent binding-inhibition titers were interpolated (Figure 7C), with the data for group 2 again showing a similar profile to those observed with the SalI variant (Figure 7D).

## Discussion

This phase Ia dose-escalation and safety study reports the first data in humans for a vaccine targeting the PvDBP_RII antigen from the blood-stage *P*. *vivax* malaria parasite. We have shown in healthy malaria-naive adult volunteers that a recombinant ChAd63-MVA heterologous prime-boost immunization regimen can induce binding-inhibitory antigen-specific serum antibody responses in addition to B and T cell responses. ChAd63 and MVA recombinant for PvDBP_RII also demonstrated a favorable safety profile. Reactogenicity of the ChAd63 PvDBP_RII vector was similar to that seen consistently with the same doses of ChAd63 vectored vaccines encoding the *P*. *falciparum* pre-erythrocytic malaria antigens ME-TRAP or PfCSP (35, 36) and the blood-stage antigens PfMSP1 or PfAMA1 (37, 38, 42, 45). In more recent years, safety and immunogenicity data for the ME-TRAP vaccines have been reported in adults, children, and infants residing in malaria-endemic areas (46). Our data with ChAd63 PvDBP_RII add to the growing body of evidence that this simian adenovirus vector is safe for clinical use. Reactogenicity of the MVA PvDBP_RII vector appeared to be more pronounced than the ChAd63 vector and increased with dose, again consistent with previous experience of using this orthopoxvirus vector for *P*. *falciparum* vaccines (37, 38) as well as other disease targets such as respiratory syncytial virus (47), hepatitis C virus (48), Ebola virus (40), HIV, and *Mycobacterium tuberculosis* (49). Indeed, the clinical safety of MVA as a recombinant vaccine vector for many infectious diseases and cancer is now well documented.

The ChAd63-MVA delivery platform was originally developed to induce T cell responses against a target blood-stage malaria antigen in humans (32, 33). Similar to our data in mice using this vaccine (27), the results presented here show that PvDBP_RII–specific IFN-γ T cell responses were induced and peaked at median levels of greater than 2,000 SFU/million PBMCs following the MVA boost. The kinetics and magnitude of this response are extremely similar to those previously seen with the same vectors encoding *P*. *falciparum* antigens (35–38). These previous studies using the ChAd63-MVA regimen, as well as other studies using alternative ChAd serotypes followed by MVA boost (40, 47, 48), have routinely shown that a mixed antigen-specific CD4^+^/CD8^+^ T cell response is induced in humans.

The ELISPOT data showed that the IFN-γ T cell responses were spread across all 6 peptide pools spanning the PvDBP_RII antigen. A previous study assessed IFN-γ and IL-10 T cell responses to aa 291–460 of PvDBP_RII in naturally exposed children and adults from Papua New Guinea. These data showed that age-dependent low-level responses are detectable in a subset of individuals following natural *P*. *vivax* infection (<150 SFU/million PBMCs using a 3-day cultured ELISPOT protocol, as opposed to the overnight stimulation used in the ex vivo assay reported here) (50). Five PvDBP_RII T cell epitopes were identified by peptide mapping, with 3 of these containing polymorphic residues leading to variant-specific cellular responses (50). Nevertheless, the contribution of T cell responses to blood-stage immunity against *P*. *vivax* remains unclear. In the case of *P*. *falciparum*, clinical trials using whole-parasite immunization (51) or ChAd63-MVA vectors encoding PfMSP1 or PfAMA1 (45) failed to show an impact on blood-stage parasite growth following CHMI despite strong T cell induction by vaccination. However, recent data from other CHMI studies show that, unlike *P*. *falciparum*, blood-stage *P*. *vivax* activates cytotoxic CD38^+^ CD8^+^ T cells that could target parasites residing within MHC class I–expressing reticulocytes (52), suggesting that it may be possible for effector T cells to play a more direct role against this species of human malaria parasite.

In agreement with preclinical data in mice and rabbits (27), the ChAd63-MVA prime-boost regimen also induced PvDBP_RII–specific serum IgG antibody responses, peaking at a median of 0.3 μg/ml after ChAd63 prime and 15.6 μg/ml after MVA boost in the full-dose vaccination groups. The kinetics and magnitude of the antigen-specific IgG, ASC, and mBC responses induced here in malaria-naive humans are consistent with those reported for the same vectors encoding the *P*. *falciparum* blood-stage antigens PfMSP1 and PfAMA1 (37, 38, 41, 42). With regard to the PvDBP_RII–specific antibody concentrations, these were lower than those seen following ChAd63-MVA immunization with PfAMA1 (37, 42) and PfMSP1 (37), but 8-fold higher than with PfCSP (36). Similar to these *P*. *falciparum* vaccines (42, 53), the anti–PvDBP_RII serum IgG response was largely composed of IgG1 and some IgG3, with moderate avidity as measured by NaSCN-displacement ELISA. These qualitative aspects of the vaccine-induced antibody responses are consistent with those observed to the same antigen following natural infection in endemic populations (54, 55); however, the contributions of antibody isotype, affinity, and avidity to protection against the *P*. *vivax* merozoite remain poorly understood.

Studies of naturally acquired immunity following *P*. *vivax* exposure have reported the induction of strain-specific immunity (43, 44) and numerous sequence polymorphisms, consistent with immune evasion, have been found within the PvDBP_RII antigen, with the majority localized to SD2 (24, 56). Nevertheless, high-titer naturally acquired BIAbs that block binding of diverse PvDBP_RII alleles from *P*. *vivax* field isolates have also been reported, albeit at low frequency (30, 31). Once acquired, these antibodies are maintained and associate with clinical immunity to *P*. *vivax*. In contrast to these epidemiological data, preclinical immunogenicity studies with the SalI allele of PvDBP_RII have shown that this immunogen is capable of eliciting high-titer, cross-reactive BIAbs, as assessed using the ELISA-based binding-inhibition assay (28). Consistent with these data, our studies here suggest that PvDBP_RII vaccination of humans can elicit antibodies that qualitatively differ from those induced by natural exposure. Across all vaccinees who received the ChAd63-MVA regimen, anti–PvDBP_RII responses were induced that blocked binding of variant PvDBP_RII alleles to DARC in vitro including one from the HMP013 strain, suggesting this strain would be suitable to test vaccine efficacy in a future phase IIa CHMI study (16). Encouragingly, median 50% binding-inhibition titers greater than 1:100 were consistently observed for all test variants; these are higher than the ~1:20 titers reported by others using a similar assay for naturally acquired, strain-transcending BIAbs in a limited number of children in Papua New Guinea and associated with clinical immunity (30). However, this association with clinical immunity has never been formally demonstrated in the context of a vaccine clinical trial. ChAd63-MVA PvDBP_RII is the first candidate vaccine against blood-stage *P*. *vivax* to reach clinical testing. It is therefore vital in a future CHMI efficacy study to assess whether vaccine-induced BIAbs associate with control of blood-stage parasite growth.

The data obtained from this study also suggested interindividual qualitative differences in terms of the polyclonal anti–PvDBP_RII IgG response, as would be anticipated following human vaccination. A recent cohort study in the Brazilian Amazon has suggested that genetic variation in HLA class II genes can influence antibody responses against PvDBP_RII following natural *P*. *vivax* infection (57). Similarly, studies of naturally acquired anti–PvDBP_RII IgG responses (58) as well as mouse monoclonal antibodies (59, 60) have reported linear and conformational epitopes. Here we failed to detect linear responses by ELISA using a peptide array, and our ongoing work will focus on elucidating epitopes recognized by vaccine-induced human B cells in order to guide future immuno-monitoring. Further ongoing work is seeking to assess antibody function against *P*. *vivax* parasites. Importantly, we have previously reported that adenovirus-MVA immunization of mice and rabbits elicits antibodies that recognize native parasite antigen by immunofluorescence assay (IFA) (27). Future studies will focus on optimizing short-term invasion–inhibition assay methodology (61) to allow for functional testing of vaccine-induced antibodies from human volunteers in clinical trials.

Overall, the association between Duffy negativity and protection against blood-stage *P*. *vivax* infection was first reported in 1976 (15), but until now this observation has not been translated into a clinical vaccine candidate. The intervening years have seen the PvDBP_RII–DARC interaction described in molecular detail and related immuno-epidemiology extensively studied in the field. Here we extend this work and demonstrate, possibly for the first time, that substantial PvDBP_RII–specific antibodies as well as B cell and T cell responses can be induced safely by immunization in humans, using a leading viral vectored delivery strategy that is in clinical development for numerous difficult and emerging diseases and cancer. Encouragingly for the *P*. *vivax* vaccine field, a second PvDBP_RII protein–based vaccine formulated in the emulsified version of glucopyranosyl lipid adjuvant (GLA-SE) has also recently entered a phase I clinical trial in India (CTRI/2016/09/007289). The demonstration in parallel of a blood-stage CHMI model for vaccine testing using *P*. *falciparum* (62), and the banking of similar blood-stage inocula for *P*. *vivax* (16), should allow for this ChAd63-MVA vaccine and others to progress to rapid phase IIa proof-of-concept efficacy testing in the near future.

## Methods

Detailed methods are provided in supplemental methods.

### ChAd63 and MVA PvDBP_RII vaccines.

The design, production, and preclinical testing of the viral vector vaccines have been reported previously (27). Briefly, both recombinant viruses express the same 984-bp coding sequence of PvDBP_RII from the SalI strain of *P*. *vivax*, aa D194–T521 (GenBank Accession DQ156512). ChAd63 PvDBP_RII was manufactured under current Good Manufacturing Practice (cGMP) conditions by the Clinical Biomanufacturing Facility (CBF), University of Oxford, UK, and MVA PvDBP_RII was manufactured under cGMP conditions by IDT Biologika GmbH, Germany, both as previously described (37).

### Study design and approvals.

The VAC051 study was a phase Ia open-label, dose-escalation, first-in-human, nonrandomized trial of the viral vectored vaccines ChAd63 PvDBP_RII and MVA PvDBP_RII given in a prime-boost regimen with an 8-week interval. The study was conducted at the Centre for Clinical Vaccinology and Tropical Medicine (CCVTM), University of Oxford, Oxford, UK. The study received ethical approval from the Oxfordshire Research Ethics Committee A in Oxford, UK (REC reference 13/SC/0001). The study was also reviewed and approved by the UK Medicines and Healthcare products Regulatory Agency (MHRA, reference 21584/0312/001-0001). Volunteers signed written consent forms and consent was verified before each vaccination. The trial was registered on Clinicaltrials.gov (NCT01816113) and was conducted according to the principles of the current revision of the Declaration of Helsinki 2008 and in full conformity with the ICH guidelines for Good Clinical Practice (GCP). The primary endpoint of the study was to assess the safety of ChAd63 PvDBP_RII and MVA PvDBP_RII, with a secondary endpoint to assess immunogenicity.

### Participants.

Healthy, malaria-naive males and nonpregnant females aged 18–50 were invited to participate in the study. All volunteers were recruited and vaccinated at the CCVTM, part of the Oxford Vaccine Centre (OVC), at the University of Oxford. Twenty-four volunteers were enrolled in total. A full list of inclusion and exclusion criteria is reported in the supplemental methods.

### Safety analysis.

Data on AEs were collected throughout a volunteer’s participation in the trial, either on the diary cards they were issued with following vaccination or at follow-up visits. Any solicited AEs occurring during the diary card period were defined as being at least possibly related to vaccination. The likely causality of all other AEs was assessed as described in the protocol and all AEs considered possibly, probably, or definitely related to vaccination are reported (Supplemental Table 1). Further details on grading are provided in the supplemental material.

### Peptides.

Peptides for ex-vivo IFN-γ ELISPOT were purchased from NEO Peptide (Supplemental Table 2), and for peptide ELISAs, biotinylated 20-mer peptides were synthesized by Mimotopes (Supplemental Table 3).

### Recombinant PvDBP_RII and DARC proteins.

Recombinant PvDBP_RII (SalI) protein for ELISA-based assays was generated using a *Drosophila melanogaster* Schneider 2 (S2) polyclonal stable cell line (ExpreS^2^ platform, ExpreS^2^ion Biotechnologies) (63). Recombinant N-terminal DARC was produced for use in the PvDBP_RII–DARC binding-inhibition assay in Oxford, UK, by transient transfection of suspension HEK293E cells grown in EXPI293 expression medium (Thermo Fisher Scientific) (34). For the HMP013 strain of *P*. *vivax*, the sequence of the PvDBP_RII gene was verified by Sanger sequencing using primers listed in Supplemental Table 4, and recombinant PvDBP_RII (HMP013) produced in suspension EXPI293F cells (Thermo Fisher Scientific) by transient transfection.

*Ex vivo IFN-γELISPOT*. Ex vivo IFN-γ ELISPOT was used to assess the kinetics and magnitude of the vaccine-induced T cell responses over time. Fresh PBMCs were used in all assays using a previously described protocol (38). Results are expressed as IFN-γ SFU per million PBMCs.

### Total IgG ELISAs.

ELISAs were performed using standardized methodology as previously described (37, 38), except that plates were coated with recombinant PvDBP_RII protein produced from the *Drosophila* S2 cells and blocked with StartingBlock T20 solution (Thermo Fisher Scientific). Responses measured in AU are reported in μg/ml following generation of a conversion factor by CFCA.

### Avidity and isotype ELISAs.

IgG antibody avidity was assessed by NaSCN-displacement ELISA using previously described methodology (53), except that plates were coated with recombinant PvDBP_RII produced from the *Drosophila* S2 cells at 2 μg/ml and blocked with StartingBlock T20 solution. The concentration of NaSCN required to reduce the OD_405_ to 50% of that without NaSCN was used as a measure of avidity (IC_50_). Antibody isotype ELISAs were also performed using methodology described in detail elsewhere (53) with the same exceptions as for the avidity ELISA.

### mBC and ASC ELISPOT.

mBC ELISPOT assays were performed as described in detail elsewhere (41). Ex vivo ASC ELISPOT assays were performed using frozen PBMCs directly prepared and added to the ELISPOT plate with no preceding 6-day culture.

### PvDBP_RII–DARC binding-inhibition assay.

Sera were tested for their ability to inhibit binding of recombinant PvDBP_RII to DARC using an assay developed at Oxford, UK (Figure 6, A and B; Figure 7, B–D; and Supplemental Figure 7A). Sera were also tested using a similar assay previously established at ICGEB, India (64) (Figures 6, C–F and Supplemental Figure 7B). Four variants of recombinant PvDBP_RII (SalI, PvAH, PvO, and PvP) were used.

### Statistics.

Data were analyzed using GraphPad Prism version 6.07 for Windows. All tests were 2-tailed and are described in the text. A value of *P* less than 0.05 was considered significant.

## Author contributions

ROP, SES, SCE, KHM, TAR, DL, ARS, GML, NJE, IDP, RF, DGWA, SCdC, AVSH, CEC, and SJD conceived and performed the experiments. ROP, SES, SCE, KHM, TAR, DL, MKH, CEC, and SJD analyzed the data. JJ, TJ, JSM, WAdJ, AN, SM, EB, and CEC contributed reagents, materials, and analysis tools. TDO assembled and annotated the genome sequence. RR and AML managed the project. ROP and SJD wrote the manuscript.

## Supplementary Material

Supplemental data

## Figures and Tables

**Table 1:**
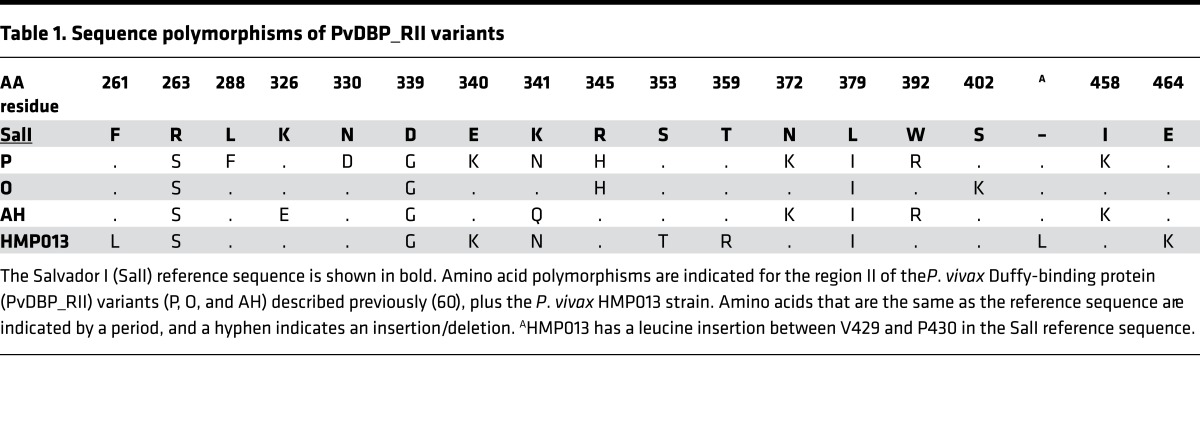
Sequence polymorphisms of PvDBP_RII variants
