# Supplementary material for: Human vaccination against Plasmodium vivax Duffy-binding protein induces strain-transcending antibodies
Source: JCI Insight. 2017 Jun 15;2(12):e93683. doi: 10.1172/jci.insight.93683 (PMC5470884; doi:10.1172/jci.insight.93683)
Supplement: Supplemental data [file jciinsight-2-93683-s001.pdf]

Supplementary Data

- Supplementary Materials and Methods
- Figure S1. Individual *ex-vivo* IFN-γ ELISPOT data.
- Figure S2. *Ex-vivo* IFN-γ ELISPOT data according to peptide pool.
- Figure S3. Individual ELISA data.
- Figure S4. Calibration-free concentration analysis.
- Figure S5. Linear peptide ELISAs.
- Figure S6. Isotype ELISA data.
- Figure S7. PvDBP_RII – DARC *in vitro* binding-inhibition.
- Table S1. Unsolicited AEs considered possibly, probably or definitely related to vaccination with ChAd63 PvDBP_RII or MVA PvDBP_RII.
- Table S2. PvDBP_RII overlapping peptides for ELISPOT assays.
- Table S3. Biotinylated PvDBP_RII overlapping peptides for ELISA assays.
- Table S4. Sequencing primers used for HMP013 PvDBP_RII.

Supplementary Materials and Methods

Study Design

The sample size for this study was chosen to allow determination of the magnitude of the outcome measures, especially of serious and severe adverse events, rather than aiming to obtain statistical significance. Four volunteers were enrolled into Groups 1 and 2A as initial safety cohorts. The decision to enrol eight volunteers into Groups 2B and 2C was based on a power calculation to have 76% power (1-β) to detect a mean two-fold improvement (two-tailed) at a significance level (α) of $P = 0.05$ in immunogenicity (IgG and T cell response) between Groups 2B and 2C following MVA PvDBP boost vaccination. Allocation to study group was based on time of enrolment and volunteer preference – Group 1 was enrolled first, followed by Groups 2A and 2B which were enrolled simultaneously, and Group 2C was the final group enrolled. There were safety reviews carried out by the Local Safety Monitor (LSM) prior to each dose escalation, and no concerns were raised with any of the vaccine doses. Participants in Groups 2B and 2C received the ChAd63 PvDBP_RII and MVA PvDBP_RII at days 0 and 56 (nominal study days are used throughout; a window period of ± 7 days was permitted in the protocol). One Group 2C participant's MVA PvDBP_RII vaccination fell outside of the window period as the participant was unwell when reviewed at the day 56 visit and the decision was made to delay vaccination for safety reasons.

Monitoring

The LSM provided safety oversight, and Good Clinical Practice compliance was independently monitored by the University of Oxford Clinical Trials and Research Governance (CTRG) Team.

Inclusion and Exclusion Criteria

A medical history and physical examination were conducted at the screening visit, as well as baseline blood tests including a full blood count; urea and electrolytes; liver function tests; and hepatitis B virus,

hepatitis C virus and human immunodeficiency virus (HIV) serology. Dipstick urinalysis for all volunteers and pregnancy testing for all female volunteers were conducted at screening. Pregnancy testing was also carried out prior to each vaccination. A full list of inclusion and exclusion criteria is shown below:

Inclusion Criteria

- Healthy adults aged 18 to 50 years;
- Able and willing (in the Investigator's opinion) to comply with all study requirements;
- Willing to allow the Investigators to discuss the volunteer's medical history with their General Practitioner;
- For females only, willingness to practice continuous effective contraception during the study and a negative pregnancy test on the day(s) of vaccination;
- Agreement to refrain from blood donation during the course of the study;
- Provide written informed consent.

Exclusion Criteria

- Participation in another research study involving receipt of an investigational product in the 30 days preceding enrolment, or planned use during the study period;
- Prior receipt of an investigational malaria vaccine or any other investigational vaccine likely to impact on interpretation of the trial data;
- Administration of immunoglobulins and/or any blood products within the three months preceding the planned administration of the vaccine candidate;
- Any confirmed or suspected immunosuppressive or immunodeficient state, including HIV infection; asplenia; recurrent, severe infections and chronic (more than 14 days) immunosuppressant medication within the past 6 months (inhaled and topical steroids are allowed);

- History of allergic disease or reactions likely to be exacerbated by any component of the vaccine, e.g. egg products, Kathon;
- History of clinically significant contact dermatitis;
- Any history of anaphylaxis in reaction to vaccination;
- Pregnancy, lactation or willingness/intention to become pregnant during the study;
- History of cancer (except basal cell carcinoma of the skin and cervical carcinoma in situ);
- History of serious psychiatric condition;
- Any other serious chronic illness requiring hospital specialist supervision;
- Suspected or known current alcohol abuse as defined by an alcohol intake of greater than 42 units every week;
- Suspected or known injecting drug abuse in the 5 years preceding enrolment;
- Seropositive for hepatitis B surface antigen (HBsAg);
- Seropositive for hepatitis C virus (antibodies to HCV) with positive PCR for hepatitis C at screening;
- History of clinical malaria (any species);
- Travel to a malaria endemic region during the study period or within the previous six months;
- Any clinically significant abnormal finding on screening biochemistry or hematology blood tests or urinalysis;
- Any other significant disease, disorder or finding which may significantly increase the risk to the volunteer because of participation in the study, affect the ability of the volunteer to participate in the study or impair interpretation of the study data;
- Inability of the study team to contact the volunteer's GP to confirm medical history and safety to participate.

Safety Analysis

Volunteers recorded their temperature daily and measured any erythema or swelling at the vaccination site for 14 days following the ChAd63 PvDBP_RII vaccination and 7 days following MVA PvDBP_RII. Any solicited AEs occurring during the diary card period were defined as being at least possibly related to vaccination. Volunteers graded all other AEs as mild, moderate or severe:

- **GRADE 0:** None.
- **GRADE 1:** Transient or mild discomfort (< 48 hours); no medical intervention/therapy required.
- **GRADE 2:** Mild to moderate limitation in activity – some assistance may be needed; no or minimal medical intervention/therapy required.
- **GRADE 3:** Marked limitation in activity, some assistance usually required; medical intervention/therapy required; hospitalization possible.

Adverse event data also included the results of the hematology (full blood count) and biochemistry (liver function tests, urea and electrolytes) carried out at all visits except those occurring 2 days post-vaccination.

The likely causality of all unsolicited AEs was assessed as per the criteria below:

- **No Relationship:** No temporal relationship to study product ***and*** alternate aetiology (clinical state, environmental or other interventions); ***and*** does not follow known pattern of response to study product.
- **Unlikely:** Unlikely temporal relationship to study product ***and*** alternate aetiology likely (clinical state, environmental or other interventions) ***and*** does not follow known typical or plausible pattern of response to study product.

- **Possible:** Reasonable temporal relationship to study product; ***or*** event not readily produced by clinical state, environmental or other interventions; ***or*** similar pattern of response to that seen with other vaccines.
- **Probable:** Reasonable temporal relationship to study product; ***and*** event not readily produced by clinical state, environment, or other interventions ***or*** known pattern of response seen with other vaccines.
- **Definite:** Reasonable temporal relationship to study product; ***and*** event not readily produced by clinical state, environment, or other interventions; ***and*** known pattern of response seen with other vaccines.

All unsolicited AEs that were assessed as being possibly, probably or definitely related to either ChAd63 PvDBP_RII or MVA PvDBP_RII are shown in Table S1.

ChAd63 and MVA PvDBP_RII Vaccines

The PvDBP_RII sequence was codon optimized for human expression and is cloned in frame at the N-terminus to the human tissue plasminogen activator (tPA) leader sequence (aa 1–32, GenBank Accession #K03021), in turn preceded by a Kozak sequence. This transgene cassette is inserted at the E1 site of the E1/E3-deleted ChAd63 vector, under the control of the 1.9 kbp CMV promoter (1). The entire E4 locus of the ChAd63 vector is also replaced with the *E4Orf6* gene from human adenovirus serotype 5. In the case of MVA, the transgene cassette is inserted at the thymidine kinase (TK) locus with expression driven by the vaccinia P7.5 early/late promoter without any additional marker (2).

Final certification of both viral vaccines and associated labelling took place at the Clinical Biomanufacturing Facility (CBF) in Oxford. Each vaccine lot underwent comprehensive quality control analysis to ensure that the purity, identity, and integrity of the virus met pre-defined specifications.

Vaccine lots were stored at the clinical site at -80 °C and the temperature was monitored. ChAd63 PvDBP_RII vaccine stability was tested using a potency assay based on *in vitro* infectivity in HEK 293 cells using anti-hexon immunostaining. MVA PvDBP_RII stability was confirmed by annual re-titrations on chick embryo fibroblast (CEF) cells and immunopotency was monitored by performing annual *in vivo* immunogenicity assays in mice.

Study Design and Approvals

Four volunteers were vaccinated with 5 x 10^9 viral particles (vp) ChAd63 PvDBP_RII (diluted in 0.9% NaCl and administered in 310 µL) in Group 1. Following a safety review, twelve volunteers were vaccinated with 5 x 10^{10} vp (given in a volume of 310 µL), seven of whom went on to receive MVA PvDBP_RII 1 x 10^8 plaque-forming units (pfu) in the opposite arm 8 weeks later (Groups 2A and 2B). One of the volunteers from Group 2B withdrew prior to MVA PvDBP_RII vaccination as they were no longer able to commit the time required for the study. A further safety review was carried out before the final eight volunteers received full dose (2 x 10^8 pfu) MVA PvDBP_RII eight weeks after vaccination with ChAd63 PvDBP_RII 5 x 10^{10} vp (Group 2C). The volumes of MVA PvDBP_RII vaccine were approximately 155 µL and 310 µL for Groups 2B and 2C respectively. All vaccinations were given intramuscularly (IM) into the deltoid muscle. Volunteers receiving two vaccinations received them in opposite arms. Volunteers in Group 1 were followed up for approximately 3 months, and volunteers in Group 2 were followed up for approximately 5 months. Volunteers attended follow-up visits on days 2, 14, 28, 56 and 84 in Group 1; days 0, 2 ,14, 28, 56, 63, 84 and 140 in Group 2A; and days 0, 2, 14, 28, 56, 58, 63, 84 and 140 in Groups 2B and 2C. Nominal study days are reported throughout. Volunteers were asked to complete diary cards for 14 days after vaccination with ChAd63 PvDBP_RII and 7 days after vaccination with MVA PvDBP_RII with details of any AEs experienced during this time. Adverse event data were also collected at all follow-up visits. Blood tests for exploratory immunology and safety (full

blood count, liver function, urea and electrolytes) were carried out at all visits after vaccination except days 2 and 58.

Participants

All volunteers signed written consent forms, and consent was checked to ensure volunteers were willing to proceed before each vaccination. Allocation to study groups (Figure 1) occurred at screening based on sequential recruitment of groups and volunteer preference.

Peripheral Blood Mononuclear Cell (PBMC) and Serum Preparation

Blood samples were collected into lithium heparin-treated vacutainer blood collection systems (Becton Dickinson, UK). PBMC were isolated and used within 6 hours in fresh assays as previously described (3). Excess cells were frozen in foetal calf serum (FCS) containing 10% dimethyl sulfoxide (DMSO) and stored in liquid nitrogen. Plasma samples were stored at -80 °C. For serum preparation, untreated blood samples were stored at room temperature (RT) and then the clotted blood was centrifuged for 5 min (1000 *xg*). Serum was stored at -80 °C.

Peptides

Peptides for *ex-vivo* IFN-γ ELISPOT were purchased from NEO Peptide (Cambridge, MA, USA). The peptides, 20 aa in length and overlapping by 10 aa, covered the entire PvDBP_RII insert present in the viral vectored vaccines (Table S2). Peptides were reconstituted in 100% DMSO at 50-200 mg/mL and combined into various pools for the ELISPOT assay. For peptide ELISAs, biotinylated 20mer peptides spanning the PvDBP_RII vaccine insert (running from residue D194–T521), each offset by 8 aa from the previous peptide (i.e. overlapping by 12 aa) (4), were synthesized by Mimotopes, Australia (Table S3). Each peptide stock was reconstituted to 50 mg/mL in DMSO.

Recombinant PvDBP_RII and DARC Proteins

Recombinant PvDBP_RII (SalI) protein for ELISA-based assays was generated as follows: The PvDBP_RII coding sequence was codon-optimized for *Drosophila* expression and synthesized (GeneArt, Life Technologies), then PCR amplified using primers 5'-CGT GGG ACT GAG TCT GGG CGA TCA CAA GAA GAC CAT CAG CAG-3' and 5'-GCT CAG GCC TCG GGC TCA TCC AGG CCC AGC AGT GGA TTG GGG ATG GTC ACG ACC TCC TGG GTG-3' leaving 19 bp 5' and 3' overhangs complementary with insertion regions of the pExpreS^2-1 expression vector (5) (ExpreS^{2}ion Biotechnologies, Denmark). Concurrently, the pExpreS^2-1 expression vector was also amplified and linearized by PCR. Plasmid cloning was done by mixing 100 ng of 1:1 molar ratio of insert:plasmid and assembled using an 8-cycle circular polymerase extension cloning (CPEC) (6) step (98 ˚C for 10 s, slow ramp anneal 70 ˚C to 55 ˚C at 0.1 ˚C/s, 72 ˚C for 35 s) using high-fidelity polymerase Phusion HF (New England Biolabs). The full open reading frame from 5' to 3' contained an 18 αα *Drosophila* binding immunoglobulin protein (BiP) leader sequence (5), αα D194–T521 of PvDBP_RII (SalI) with T257A, S353A and T422A substitutions to remove sites of possible N-linked glycosylation, followed by a shortened 9 αα Pk/V5 epitope tag (IPNPLLGLD) and a 4 αα C-tag (EPEA) (7) for detection and purification, respectively. Nicked plasmids were transformed into competent DH5α *Escherichia coli* and selected for using the Zeocin resistance marker. This plasmid was used to generate a *Drosophila melanogaster* Schneider 2 (S2) polyclonal stable cell line using the ExpreS^2 platform as previously described (5) except that 2 mg/mL zeocin was used for selection, prior to growing the cell line also as previously described (5). Clarified supernatant from a 4-day batch culture was concentrated 15-20 fold and buffer exchanged using a Tangential Flow Filtration (TFF) system fitted with Pellicon 3 Ultracel 10 kDa membrane (Merck Millipore, UK). Purification was performed on an AKTA Pure 25 system (GE Healthcare, UK), consisting of an affinity step with CaptureSelect™ C-tag column (Thermo Fisher Scientific, UK) and a polishing size exclusion chromatography (SEC) using Superdex 200 16/60 PG (GE

Healthcare, UK) in 20 mM Tris-HCl, 150 mM NaCl, pH 7.4 (TBS). Purified protein was quantified by Nanodrop (Thermo Fisher Scientific, UK) and stored at -80 °C until further use.

Recombinant N-terminal DARC was produced for use in the PvDBP_RII – DARC binding-inhibition assay in Oxford, UK. A gene encoding the first 60 αα of DARC Fyb allele (GenBank Accession # ABA10433.1) followed by a thrombin cleavage site (LVPRGS) and an AviTag (GLNDIFEAQKIEWHE) was codon-optimized for *E. coli* expression and synthesized (GeneArt, Life Technologies). Cysteines 4, 51 and 54 of DARC were mutated to alanine (8, 9). This sequence with 5' KpnI site and 3' BamHI site was cloned into a mammalian expression plasmid (10) in-frame with an N-terminal human tissue plasminogen activator (tPA) leader sequence (11) and a C-terminal hexa-histidine tag prior to the stop codon. Suspension HEK293E cells grown in EXPI293 expression medium (Thermo Fisher Scientific, UK) were transiently transfected with plasmid (10) and allowed to grow for three days before the supernatant was harvested, purified using a HisTrap Excel column (GE Healthcare, UK) and buffer exchanged into PBS. Purified protein was quantified by Nanodrop (Thermo Fisher Scientific, UK) and stored at -80 °C until further use.

For the HMP013 strain, 200 mL of *P. vivax*-infected was collected from a patient with vivax malaria (12), with ethical approval from the Royal Brisbane and Women's Hospital (reference 10/QRBW/379). The blood was leukodepleted by passage through an inline white cell trap (Pall Medical) and parasite DNA extracted using a Qiagen Blood Midi kit. Whole genome sequencing was performed using an Illumina MiSeq 150 bp reads. The reads were assembled with MaSurRCA (13) and, after removal of 16 contigs containing human contamination (BLASTn), we obtained an assembly of 538 scaffolds with a total size of 30.6 Mb. Scaffolds were ordered with ABACAS (14) against the *P. vivax* reference PvP01 (15) and annotated with Companion (16), calling 7032 genes (including pseudo genes). The raw sequencing reads and the assembly are deposited with the project accession ERP021294. The annotation and sequence can

also be found at ftp://ftp.sanger.ac.uk/pub/project/pathogens/Plasmodium/P_vivax/PvHMP-013. To verify the sequence of the PvDBP gene, individual segments of the PvDBP_RII coding sequence were PCR amplified and sequenced using standard Sanger sequencing methods using primers listed in Table S4. The full-length PvDBP_RII sequence was assembled from these amplicons and aligned to the reference sequence in NCBI. Recombinant PvDBP_RII (HMP013) was subsequently produced for use in the PvDBP_RII – DARC binding-inhibition assay in Oxford, UK. A gene encoding PvDBP_RII (aa 194-521) was codon-optimized for human expression and synthesized (Genewiz, USA). This sequence, with a 5' KpnI site and 3' XbaI site, was cloned into a mammalian expression plasmid (10) in frame with an N-terminal mouse IgK light chain leader sequence and a C-terminal C-tag (7) prior to the stop codon. Suspension EXPI293F cells (Thermo Fisher Scientific, UK) were transiently transfected and culture supernatants were harvested after 4 days. Purification was performed on an AKTA Pure 25 system (GE Healthcare, UK), consisting of an affinity step with CaptureSelect™ C-tag column (Thermo Fisher Scientific, UK) and a polishing SEC using Superdex 200 Increase 10/300 GL (GE Healthcare, UK) in 20 mM Tris-HCl, 150 mM NaCl, 0.1% Tween 20, pH 7.4 (TBS). Purified protein was quantified by Nanodrop (Thermo Fisher Scientific, UK) and stored at -80 °C until further use.

Ex-vivo IFN-γ ELISPOT

Fresh PBMC were used in all assays using a previously described protocol (3), except that 50 µL/well PvDBP_RII peptide pools (Table S2) (final concentration each peptide 5 µg/mL) were added to triplicate test wells, 50 µL/well R10 and DMSO control were added to negative unstimulated wells, and 50 µL/well Staphylococcal enterotoxin B (SEB) (final concentration 0.02 µg/mL) plus phytohemagglutinin (PHA) (final concentration 10 µg/mL) was added to positive control wells. Spots were counted using an ELISPOT counter (Autoimmun Diagnostika (AID), Germany). Results are expressed as IFN-γ spot-forming units (SFU) per million PBMC. Background responses in unstimulated control wells were almost always less than 20 spots, and were subtracted from those measured in peptide-stimulated wells.

Total IgG and Peptide ELISAs

ELISAs were performed using the standardized methodology as previously described (3, 17), except that plates were coated with recombinant PvDBP_RII protein produced from the *Drosophila* S2 cells at a concentration of 2 µg/mL in PBS and left over-night and the blocking step used Starting Block T20 solution (Fisher, UK). The reference serum used to generate the standard curve was prepared from a high-responding Group 2C volunteer's day 84 serum sample. The reciprocal of the dilution giving an optical density at 405nm (OD_{405}) of 1.0 in the standardized assay was used to assign an ELISA unit value of the standard. The standard curve and Gen5 ELISA software v2.07 (BioTek, UK) was used to convert the OD_{405} of individual test samples into arbitrary units (AU). These responses in AU are reported in µg/mL following generation of a conversion factor by calibration-free concentration analysis (CFCA).

For peptide ELISAs, 96 well Nunc-Immuno Maxisorp plates were coated with individual peptides at a concentration of 10 µg/mL in PBS and left at 4 °C over-night. Plates were then washed with PBS containing 0.05% Tween 20 (PBS/T) x6 and blocked for 1 h with Casein block solution (Pierce). After another wash step, test plasma diluted 1:100 in casein block solution were added to the plates for 2 h. Plates were washed again and alkaline phosphatase-conjugated goat anti-human IgG (γ-chain) (Sigma) diluted 1:1000 in casein block solution was added, before development with *p*-nitrophenylphosphate substrate (Sigma) diluted in diethanolamine buffer (Thermo Fisher Scientific). OD_{405} was read using an Infinite F50 microplate reader (Tecan) and Magellan v7.0 software.

Calibration-Free Concentration Analysis (CFCA)

Anti-PvDBP_RII total IgG ELISA AU were converted to antigen-specific IgG concentration in µg/mL as follows. CFCA was performed with a method similar to that previously described (18, 19), using a

Biacore X100 instrument, a Biotin CAP chip, and X100 control and evaluation software (GE Lifesciences, UK). Mono-biotinylated PvDBP_RII supernatant was produced for use in the CFCA by transient transfection of suspension EXPI293F cells, using a plasmid encoding PvDBP_RII (SalI) with C-terminal rat CD4 domains 3 and 4 followed by a biotin acceptor peptide obtained from Addgene (plasmid #68529) and courtesy of Dr Julian Rayner (Wellcome Trust Sanger Institute, Hinxton, UK) (20). This PvDBP_RII plasmid was co-transfected with another plasmid encoding *E. coli* biotin ligase (BirA). Supernatant was harvested after 4 days, clarified, dialysed against PBS using snakeskin and concentrated ~10-fold using spin columns. CFCA was subsequently performed using day 84 plasma samples from Group 2C individuals with a range of PvDBP_RII-specific IgG antibody responses as assessed by ELISA. Each individual's plasma was diluted 1:100 in running buffer (18) and assayed at least twice on different days. The results were very similar between independent runs and a single dataset was chosen for final analysis (Figure S4). Mass-transport limited binding conditions were obtained by capturing a minimum of 5000 response units (RU) of PvDBP_RII antigen on the active flow cell. The chip was regenerated with the manufacturer's supplied regeneration and CAP reagents (diluted 1:2 in HBS EP+) and fresh antigen (neat supernatant) prior to each application of antibody; variation in the level of antigen capture between cycles was typically <2%. Antigen-specific antibody binding was measured by double reference subtraction, firstly of binding to a flow cell coated only with the biotin capture reagent, and secondly of the binding of a day 0 plasma sample (from the same volunteer) from that of the day 84 sample (Figure S4A). Initial rates of antigen-specific binding at 5 µL/min and 100 µL/min were measured and compared to permit measurement of concentration and the level of mass-transport limitation. Parameters (initial binding rate >0.3 RU/s at 5 µL/min flow rate and >0.13 for quality control (QC) ratio) recommended by the manufacturer were used for quality control of data (Figure S4B). Initial binding rates were in the range 0.33 – 1.1 RU/s at 5 µL/min flow, and calculated QC ratios were all >0.24 (reflecting adequate mass transport limitation for concentration estimation). The binding model used a molecular weight of 150 kDa for IgG. The viscosity of the running buffer at 20 °C was 1.0562 mPa*s (measured using a

densitometer equipped with a viscometer module) giving a measured diffusion coefficient of IgG at 20 °C in a solution with the viscosity of water, pH 7.4, of 4.8 $x10^{-11}$ m^2/s. The diffusion coefficient of IgG under the test conditions (25 °C) was therefore calculated to be 5.494 x 10^{-11} m^2/s. The CFCA-measured PvDBP_RII-specific antibody concentrations for each individual were analysed by linear regression with the corresponding total IgG ELISA AU data, with the slope of the line used to derive an AU-to-µg/mL conversion factor.

Memory B cell and ASC ELISPOT

B cell ELISPOT assays were performed as described in detail elsewhere (21). In brief, to measure mBC responses, frozen PBMC were thawed before culturing with a polyclonal B cell stimulation mix containing *Staphylococcus aureus* Cowan strain Pansorbin cell 'SAC' (Calbiochem), the human TLR agonist CpG ODN-2006 (Invivogen) and pokeweed mitogen 'PWM' (Sigma) for 6 days, allowing mBC to differentiate into ASC. On day five of the experiment, ELISPOT plates were coated with recombinant PvDBP_RII protein produced from the *Drosophila* S2 cells to measure the antigen-specific response and polyvalent goat-anti human IgG (Caltag) to measure the total IgG response. PBS coated wells were used as a negative control. On day six, cultured cells were transferred to the ELISPOT plate and incubated for 18-20 h before developing with an anti-human IgG (γ-chain) antibody conjugated to alkaline phosphatase (Calbiochem) followed by a substrate buffer. Plates were counted using an AID ELISPOT plate reader. *Ex-vivo* ASC ELISPOT assays were performed exactly as above but using frozen PBMC directly prepared and added to the ELISPOT plate with no preceding 6 day culture.

PvDBP_RII – DARC Binding-Inhibition Assay

Sera were tested for their ability to inhibit binding of recombinant PvDBP_RII with DARC using an assay developed at Oxford, UK (Figure 6A,B, Figure 7B-D and Figure S7A). Recombinant N-terminal

DARC protein was coated onto Nunc-Immuno Maxisorp plates at 1 µg/mL in Dulbecco's PBS (DPBS – Sigma Aldrich, UK) overnight at 4 °C. Plates were washed with PBS and 0.05 % Tween 20 (PBS/T) and blocked with 2 % skimmed milk in PBS for 2 h at 37 °C. Day 84 serum samples were diluted in a 2-fold series (from 1:5 to 1:640) in 0.25 % milk in PBS, and for each test serum sample a matched pre-immune (d0) serum from the same individual was assayed at a dilution of 1:5. 50 µL of each test sample (added at twice the final desired concentration) was then pre-incubated with 50 µL of the relevant test variant of PvDBP_RII (at a concentration 0.1 µg/mL in 0.25 % skimmed milk in PBS) for 30 min at RT. The pre-incubated PvDBP_RII protein plus serum was then added to the DARC-coated plates (in duplicate wells). The plates were then incubated for 1 h at 37 °C, washed with PBS/T and incubated with anti-PvDBP_RII polyclonal rabbit serum (11) diluted 1:1000 in 0.25 % milk in PBS. After another wash in PBS/T, plates were incubated with a 1:1000 dilution of anti-rabbit IgG alkaline phosphatase (Sigma Aldrich, UK) to detect bound PvDBP_RII protein. After a final wash with PBS/T, the plates were developed with 1 mg/mL *p*-nitrophenylphosphate in diethanolamine buffer (Pierce, UK) as developing substrate. OD_{405} was read using a Model 550 Microplate Reader (Bio-Rad, UK) when the wells containing PvDBP_RII protein and buffer only (no serum) reached a value of 1.0. In one column of each 96 well plate, wells contained only day 84 serum (diluted 1:5) and buffer (no PvDBP_RII protein) and these 'background' OD_{405} values were subtracted from all test values (both d84 and d0). Subsequently to calculate % binding-inhibition for each d84 test sample we used the formula:

$$1 - (OD_{405} \text{ value of d84 serum sample} / OD_{405} \text{ value of corresponding d0 serum sample}) \times 100\ \%$$

Sera were also tested for their ability to inhibit binding of PvDBP_RII with DARC using a similar assay previously established at ICGEB, India (22) (Figures 6C-F and Figure S7B). Four variants of recombinant PvDBP_RII (SalI, PvAH, PvO and PvP) were used. The PvO sequence is the same as that recently reported in the new *P. vivax* reference strain PvP01 (15). DARC-Fc was coated on ELISA plates at 1 µg/mL overnight at 4 °C. Plates were washed with PBS/T and blocked with 2% skimmed milk in PBS for

2 h at 37 °C. Serum samples were diluted in series (1:10, 1:50, 1:100, 1:500 and 1:1,000) and each serum dilution was pre-incubated with the relevant test variant of PvDBP_RII (0.025 µg/mL) for 1 h at RT. Pre-incubated PvDBP_RII protein plus serum was then added to the DARC-coated plates (in duplicate/triplicate wells). The relevant test PvDBP_RII protein variant was also added on each plate, in a series of concentrations (0 – 0.025 µg/mL), to generate a PvDBP_RII binding standard curve. Plates were incubated for 1 h at 37 °C. Plates were then washed and incubated with anti-PvDBP_RII polyclonal rabbit antibody (22), followed by HRP-conjugated anti-rabbit IgG (whole molecule) antibody (Sigma) to detect bound PvDBP_RII. Plates were developed with o-phenylenediamine dihydrochloride (OPD) substrate (Sigma) and optical density measured at 492 nm using SoftMax Pro software, that interpreted the OD values as concentrations of bound PvDBP_RII based on the standard curve on each plate. Percent inhibition at each dilution was determined as (100 % – % binding). A curve of % inhibition versus serum dilution was used to determine 50% binding-inhibition titer of each sample.

Supplementary Figures

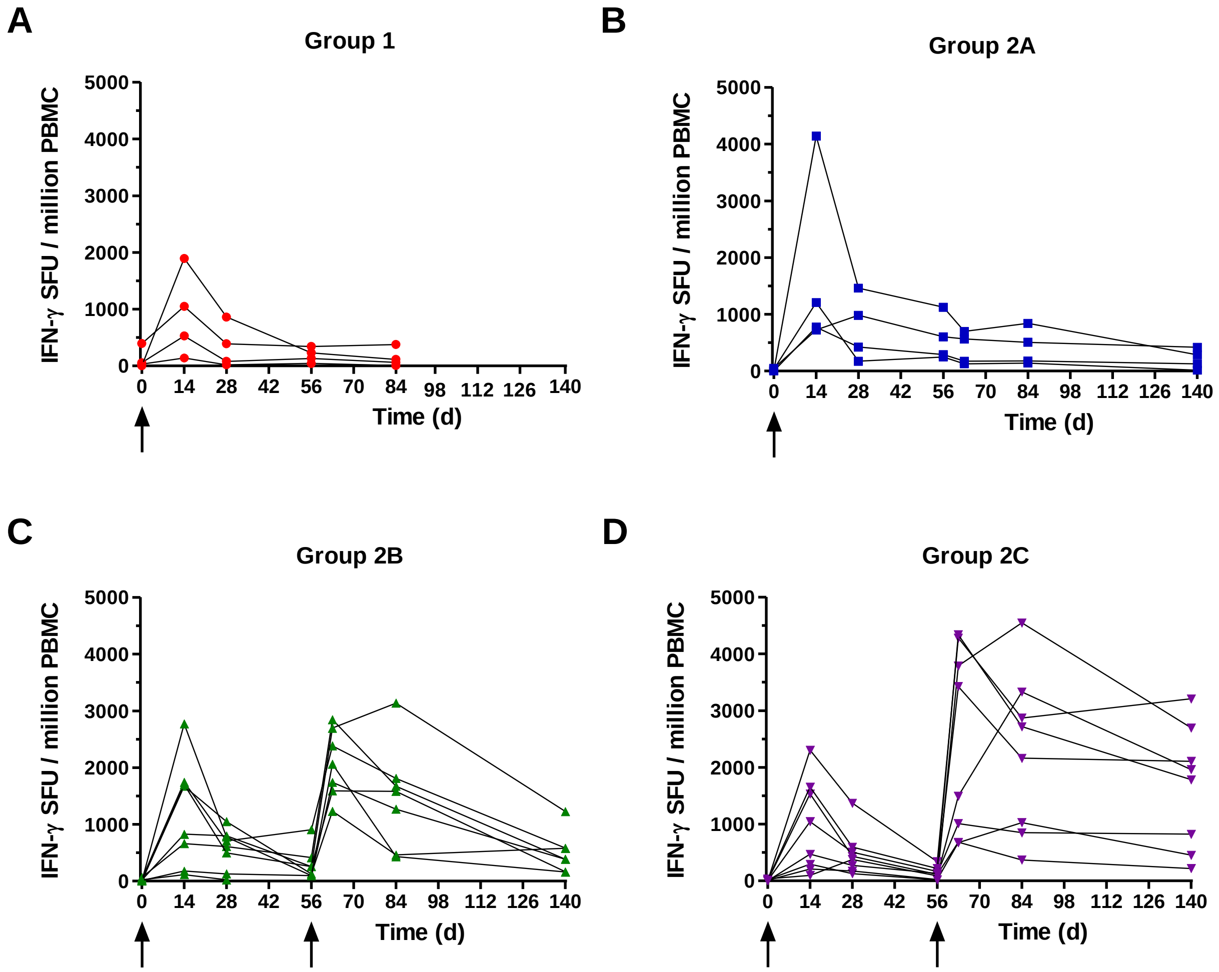

Supplementary Figure 1. Individual *ex-vivo* IFN-γ ELISPOT data.

ELISPOT responses to the PvDBP_RII insert (summed response across all the individual peptide pools) are shown over time following immunization in **(A)** Group 1 (n=4); **(B)** Group 2A (n=4); **(C)** Group 2B (n=8 until d28, and then n=7 from d56 onwards); and **(D)** Group 2C (n=8). Individual responses are shown for each volunteer.

A

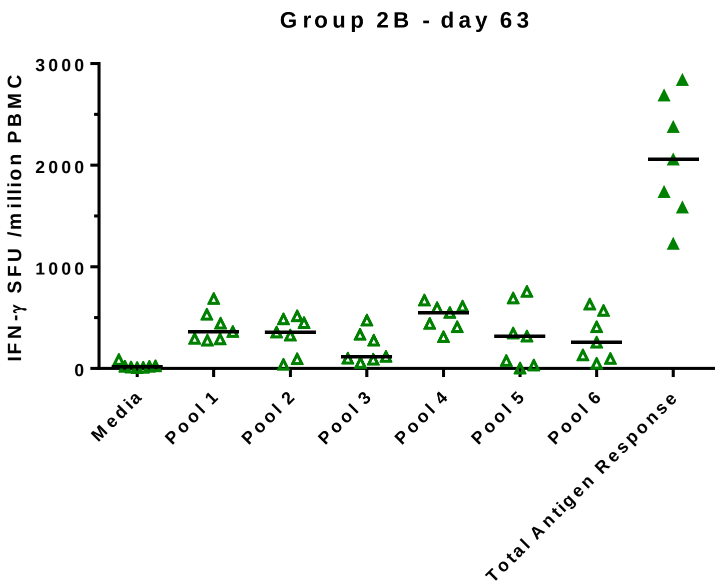

B

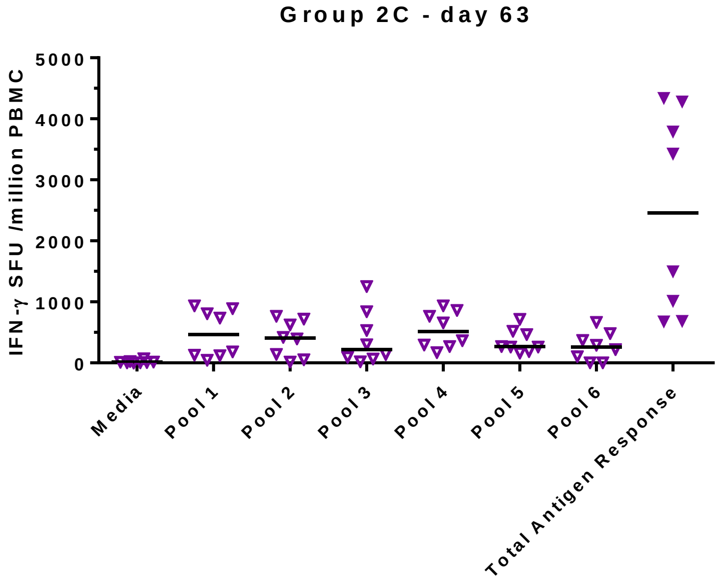

Supplementary Figure 2. *Ex-vivo* IFN-γ ELISPOT data according to peptide pool.

ELISPOT responses to the PvDBP_RII insert at day 63 for Groups 2B and 2C are shown according to the six peptide pools used in the assay (Table S2). Individual and median responses are shown for each pool, the media control and the total summed response.

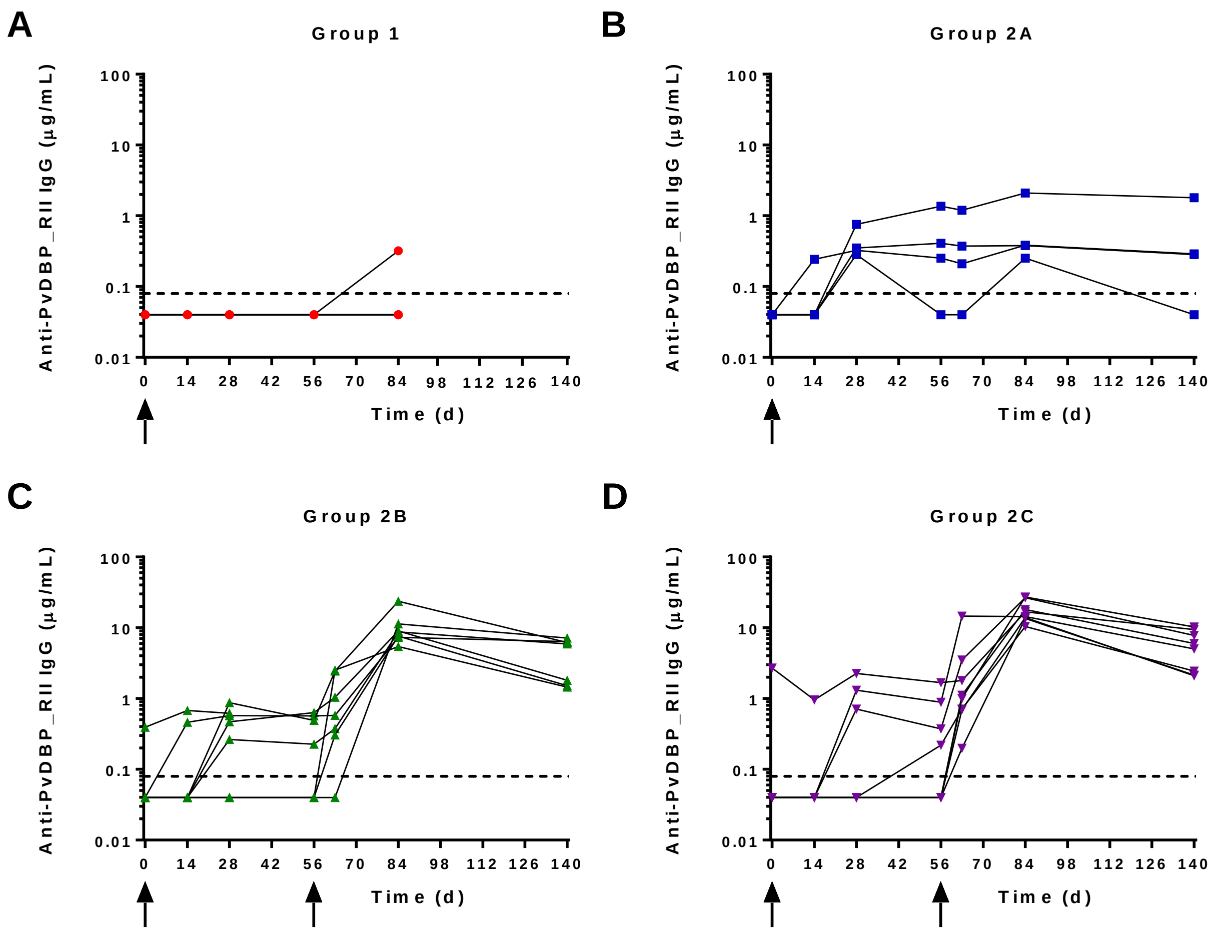

Supplementary Figure 3. Individual ELISA data.

Serum total IgG ELISA responses to recombinant PvDBP_RII are shown over time following immunization in **(A)** Group 1 (n=4); **(B)** Group 2A (n=4); **(C)** Group 2B (n=8 until d28, and then n=7 from d56 onwards); and **(D)** Group 2C (n=8). The horizontal dotted line indicates the limit of detection of the assay. Individual responses are shown for each volunteer. Two volunteers (one in Group 2B and one in Group 2C), showed responses above background at baseline (day 0).

A

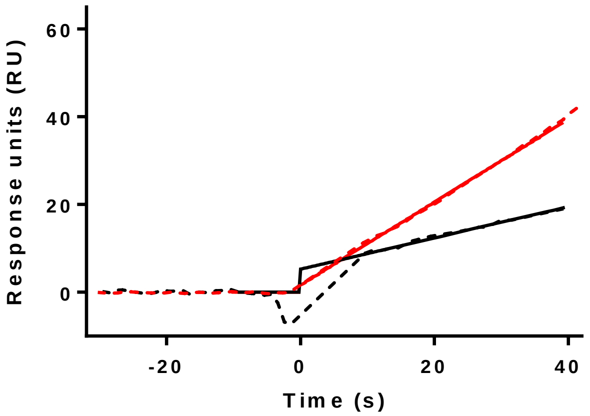

B

Sample	Calculated Concentration (µg/mL)	Fitted initial binding rate (RU/s)		QC ratio fit	ELISA (AU)
		5 µL/min flow	100 µL/min flow		
**G2C d84 #1**	6.8	0.36	0.94	0.97	2612
**G2C d84 #2**	6.4	0.34	0.91	0.99	4543
**G2C d84 #3**	11.1	0.51	1.15	0.72	3387
**G2C d84 #4**	9.9	0.52	1.40	0.99	3481
**G2C d84 #5**	22.0	0.99	2.13	0.67	6810
**G2C d84 #6**	26.7	1.08	2.09	0.54	6667
**G2C d84 #7**	15.0	0.65	1.36	0.63	3589

C

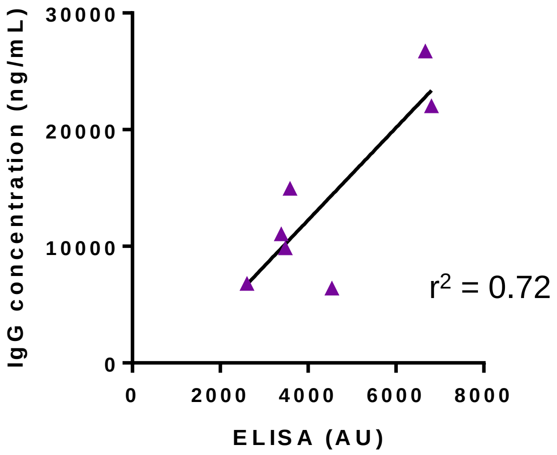

Conversion factor (slope)

4.0 (95% CI: 1.1 – 6.8) ng/mL

per 1 AU (ELISA unit)

Supplementary Figure 4. Calibration-free concentration analysis (CFCA).

CFCA was performed using a Biacore X100 instrument in order to measure absolute (µg/mL) concentrations of PvDBP_RII-specific antibody in day 84 plasma samples from 7 subjects in Group 2C.

(**A**) Binding at low sample flow rate (5 µL/min) is indicated in black while binding at high flow (100 µL/min) is indicated in red. Example data are shown for one test d84 sample with the graph showing final double-subtracted PvDBP_RII-specific binding at the two flow rates. Day 84 sample binding to the non-PvDBP_RII coated reference flow cell (Fc1) was subtracted from the PvDBP_RII coated active flow cell (Fc2), followed by subtraction of volunteer-matched pre-immune d0 plasma binding (also calculated as Fc2-Fc1). Dashed lines show the test sample data, and the solid lines the fitted data generated by X100 evaluation software. The slopes of these solid lines were used to calculate antigen-specific antibody concentration in the test d84 sample. (**B**) Tabulates CFCA and relevant ELISA results for the 7 samples. (**C**) Correlation of ELISA AU for each sample and the PvDBP_RII-specific IgG concentration measured by CFCA. Linear regression r^2 value is shown; with the slope used to define the conversion factor between ELISA AU and antigen-specific IgG concentration in ng/mL.

A

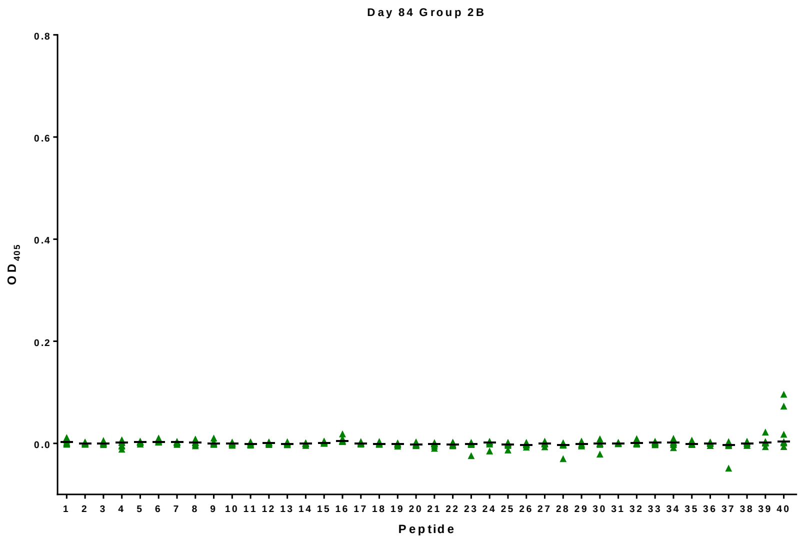

B

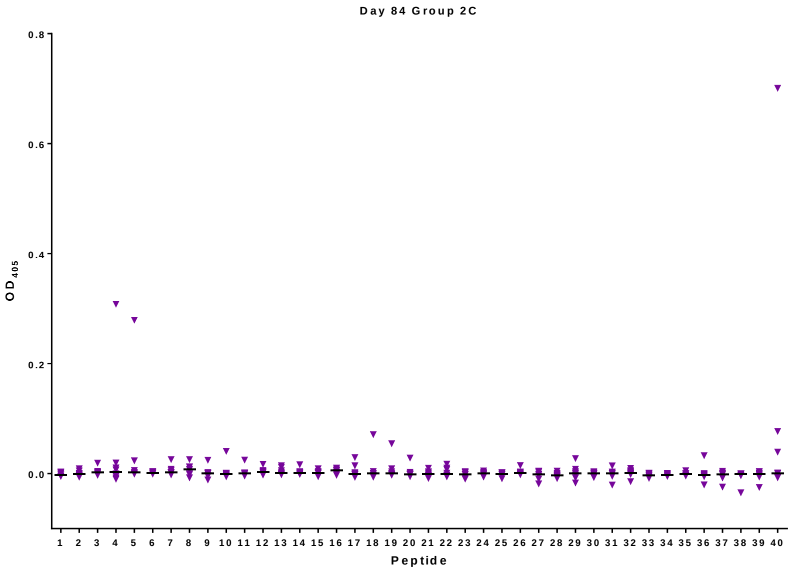

Supplementary Figure 5. Linear peptide ELISAs.

Day 0 and day 84 plasma for volunteers in (**A**) Group 2B (n=7) and (**B**) Group 2C (n=8) were diluted 1:100 and tested against linear overlapping peptides spanning the PvDBP_RII vaccine insert. OD_{405} readings for day 0 samples were all typically < 0.1 and this background response to each peptide was subtracted from the day 84 OD_{405} reading for the corresponding peptide. Individual and median results are shown.

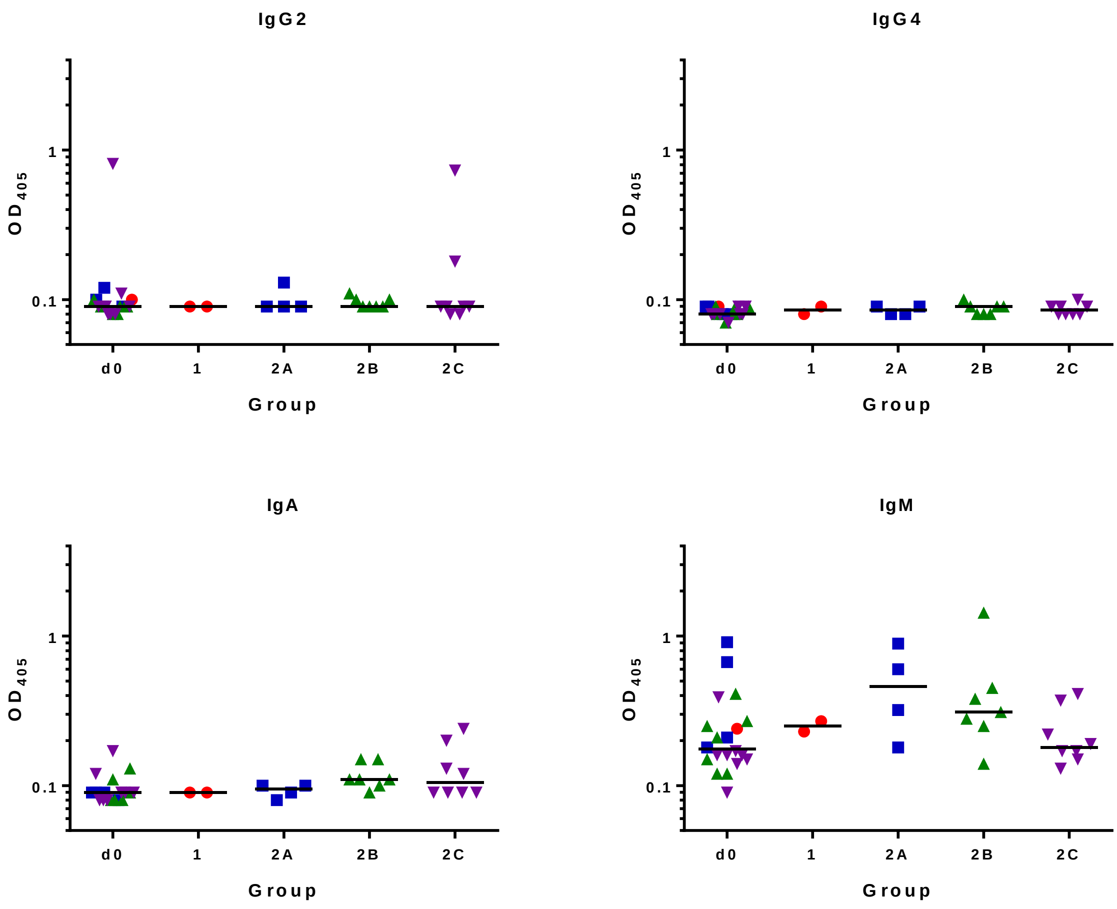

Supplementary Figure 6. Isotype ELISA data.

Isotype profiles of serum antibody responses were assessed by ELISA. Responses are shown at baseline (d0) and for all groups at day 84. Individual and median responses are shown for IgG2, IgG4, IgA and IgM. IgG1 and IgG3 are shown in Figure 4E.

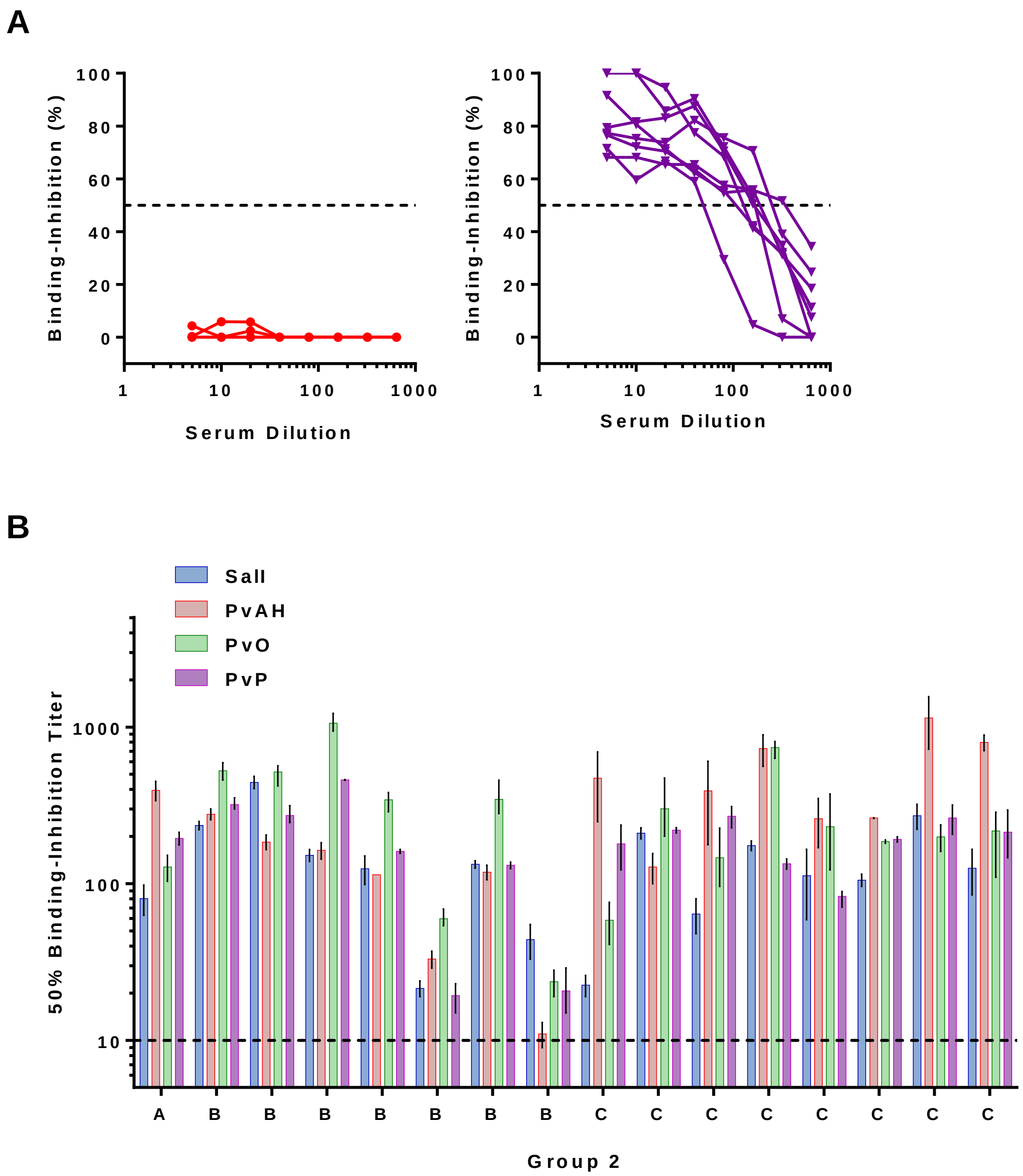

Supplementary Figure 7. PvDBP_RII – DARC *in vitro* binding-inhibition.

(**A**) Day 84 sera were tested for their ability to inhibit binding of recombinant PvDBP_RII (SalI) to DARC using an ELISA-based assay in Oxford. Samples were titrated starting at 1:5 dilution down to 1:640. Dashed line indicates 50% binding-inhibition. Data are shown for Group 1 (red) and Group 2C (purple). (**B**) Day 0 and day 84 sera were assessed as in (**A**) using the assay established at ICGEB, India,

using four recombinant alleles of PvDBP_RII: SalI, PvAH, PvO and PvP. Results are shown for each serum sample giving a positive response (d84 samples only for one volunteer in Group 2A, and all volunteers in Groups 2B and 2C). Bars show the mean result ± range of each sample tested in duplicate or triplicate.

Supplementary Tables

**Unsolicited AEs post-ChAd63 PvDBP_RII 5 x 10^9 vp**	**AE start day**	**Severity**
Bad dreams overnight	1	Mild
General malaise following lack of sleep and drinking alcohol	2	Mild
**Unsolicited AEs post-ChAd63 PvDBP_RII 5 x 10^{10} vp**	**AE start day**	**Severity**
Arthralgia in hip area	0	Mild
Blocked nose	0	Mild
Bruising at site of vaccination	1	Mild
Coryzal symptoms	1	Mild
**Unsolicited AEs post-MVA PvDBP_RII 1 x 10^8 pfu**	**AE start day**	**Severity**
Bruise at vaccine site	0	Mild
**Unsolicited AEs post-MVA PvDBP_RII 2 x 10^8 pfu**	**AE start day**	**Severity**
Sore throat	0	Mild
Dry cough	1	Mild
5 mm diameter red mark 2 inches from vaccination site, felt warm	1	Mild
Slight tightness of the chest	5	Mild
Raised lymph nodes in neck	3	Mild

Supplementary Table 1. Unsolicited AEs considered possibly, probably or definitely related to vaccination with ChAd63 PvDBP_RII or MVA PvDBP_RII.

#	aa Sequence	Pool
**PvD1**	DHKKTISSAIINHAFLQNTV	1
**PvD2**	INHAFLQNTVMKNCNYKRKR	1
**PvD3**	MKNCNYKRKRRERDWDCNTK	1
**PvD4**	RERDWDCNTKKDVCIPDRRY	1
**PvD5**	KDVCIPDRRYQLCMKELTNL	1
**PvD6**	QLCMKELTNLVNNTDTNFHR	1
**PvD7**	VNNTDTNFHRDITFRKLYLK	2
**PvD8**	DITFRKLYLKRKLIYDAAVE	2
**PvD9**	RKLIYDAAVEGDLLLKLNNY	2
**PvD10**	GDLLLKLNNYRYNKDFCKDI	2
**PvD11**	RYNKDFCKDIRWSLGDFGDI	2
**PvD12**	RWSLGDFGDIIMGTDMEGIG	3
**PvD13**	IMGTDMEGIGYSKVVENNLR	3
**PvD14**	YSKVVENNLRSIFGTDEKAQ	3
**PvD15**	SIFGTDEKAQQRRKQWWNES	3
**PvD16**	QRRKQWWNESKAQIWTAMMY	3
**PvD17**	KAQIWTAMMYSVKKRLKGNF	4
**PvD18**	SVKKRLKGNFIWICKLNVAV	4
**PvD19**	IWICKLNVAVNIEPQIYRWI	4
**PvD20**	NIEPQIYRWIREWGRDYVSE	4
**PvD21**	REWGRDYVSELPTEVQKLKE	4
**PvD22**	LPTEVQKLKEKCDGKINYTD	5
**PvD23**	KCDGKINYTDKKVCKVPPCQ	5
**PvD24**	KKVCKVPPCQNACKSYDQWI	5
**PvD25**	NACKSYDQWITRKKNQWDVL	5
**PvD26**	TRKKNQWDVLSNKFISVKNA	5
**PvD27**	SNKFISVKNAEKVQTAGIVT	6
**PvD28**	EKVQTAGIVTPYDILKQELD	6
**PvD29**	PYDILKQELDEFNEVAFENE	6
**PvD30**	EFNEVAFENEINKRDGAYIE	6
**PvD31**	INKRDGAYIELCVCSVEEAK	6
**PvD32**	LCVCSVEEAKKNTQEVVT	6

Supplementary Table 2. PvDBP_RII overlapping peptides for ELISPOT assays.

20mer peptides overlapping by 10 aa were generated for the whole of the PvDBP_RII vaccine insert present in the ChAd63 and MVA vaccines (except for the final peptide PvD32 which is an 18mer overlapping by 10aa). Peptide sequences are shown, and pools used in the ELISPOT assay indicated.

#	N-term	Sequence	C-term
**1**	Amine	DHKKTISSAIINHAFLQNTVGSG	Lys(Biotin)-Amide
**2**	Biotin-	SGSGAIINHAFLQNTVMKNCNYKR	-Amide
**3**	Biotin-	SGSGQNTVMKNCNYKRKRRERDWD	-Amide
**4**	Biotin-	SGSGNYKRKRRERDWDCNTKKDVC	-Amide
**5**	Biotin-	SGSGRDWDCNTKKDVCIPDRRYQL	-Amide
**6**	Biotin-	SGSGKDVCIPDRRYQLCMKELTNL	-Amide
**7**	Biotin-	SGSGRYQLCMKELTNLVNNTDTNF	-Amide
**8**	Biotin-	SGSGLTNLVNNTDTNFHRDITFRK	-Amide
**9**	Biotin-	SGSGDTNFHRDITFRKLYLKRKLI	-Amide
**10**	Biotin-	SGSGTFRKLYLKRKLIYDAAVEGD	-Amide
**11**	Biotin-	SGSGRKLIYDAAVEGDLLLKLNNY	-Amide
**12**	Biotin-	SGSGVEGDLLLKLNNYRYNKDFCK	-Amide
**13**	Biotin-	SGSGLNNYRYNKDFCKDIRWSLGD	-Amide
**14**	Biotin-	SGSGDFCKDIRWSLGDFGDIIMGT	-Amide
**15**	Biotin-	SGSGSLGDFGDIIMGTDMEGIGYS	-Amide
**16**	Biotin-	SGSGIMGTDMEGIGYSKVVENNLR	-Amide
**17**	Biotin-	SGSGIGYSKVVENNLRSIFGTDEK	-Amide
**18**	Biotin-	SGSGNNLRSIFGTDEKAQQRRKQW	-Amide
**19**	Biotin-	SGSGTDEKAQQRRKQWWNESKAQI	-Amide
**20**	Biotin-	SGSGRKQWWNESKAQIWTAMMYSV	-Amide
**21**	Biotin-	SGSGKAQIWTAMMYSVKKRLKGNF	-Amide
**22**	Biotin-	SGSGMYSVKKRLKGNFIWICKLNV	-Amide
**23**	Biotin-	SGSGKGNFIWICKLNVAVNIEPQI	-Amide
**24**	Biotin-	SGSGKLNVAVNIEPQIYRWIREWG	-Amide
**25**	Biotin-	SGSGEPQIYRWIREWGRDYVSELP	-Amide
**26**	Biotin-	SGSGREWGRDYVSELPTEVQKLKE	-Amide
**27**	Biotin-	SGSGSELPTEVQKLKEKCDGKINY	-Amide
**28**	Biotin-	SGSGKLKEKCDGKINYTDKKVCKV	-Amide
**29**	Biotin-	SGSGKINYTDKKVCKVPPCQNACK	-Amide
**30**	Biotin-	SGSGVCKVPPCQNACKSYDQWITR	-Amide
**31**	Biotin-	SGSGNACKSYDQWITRKKNQWDVL	-Amide
**32**	Biotin-	SGSGWITRKKNQWDVLSNKFISVK	-Amide
**33**	Biotin-	SGSGWDVLSNKFISVKNAEKVQTA	-Amide
**34**	Biotin-	SGSGISVKNAEKVQTAGIVTPYDI	-Amide
**35**	Biotin-	SGSGVQTAGIVTPYDILKQELDEF	-Amide
**36**	Biotin-	SGSGPYDILKQELDEFNEVAFENE	-Amide
**37**	Biotin-	SGSGLDEFNEVAFENEINKRDGAY	-Amide
**38**	Biotin-	SGSGFENEINKRDGAYIELCVCSV	-Amide
**39**	Biotin-	SGSGDGAYIELCVCSVEEAKKNTQ	-Amide
**40**	Biotin-	SGSGIELCVCSVEEAKKNTQEVVT	-Acid

Supplementary Table 3. Biotinylated PvDBP_RII overlapping peptides for ELISA assays.

20mer peptides overlapping by 12 aa were generated for the whole of the PvDBP_RII vaccine insert present in the ChAd63 and MVA vaccines. Underlined aa sequences were included as linkers between the biotin and 20mer. Chemistries at the two termini are also indicated.

Primer	5' – 3' Sequence
**SalIF**	GTA CAT CGC TTT CTG C
**SalIR**	ATC AAT TTA CGG GGC G
**SousaNew-Rev**	ATT CGT AAG TTC CTT CAT ACA T
**DBL-3'Re-For**	ACC TGC CGT CTG AAC CT
**PvDBPFwd**	ATT CGT AAG TTC CTT CAT ACA T
**PvDBPRev**	ACC TGC CGT CTG AAC CTT TT
**DBL.RF#2-F**	TTC CAC CAC TGT TTA CGA C
**DBL.RF#2-R**	CCA TTG CTA GAT GTC TCA TAA
**DBL.RF#3-F**	TTG ACA TGG TGG TAC CTT A
**DBL.RF#3-R**	TTA TGA GAC ATC TAG CAA TGG
**DBL.RF#4-F**	TTC GGC AAT ATC ACT TCG
**DBL.RF#4-R**	GAC ATG GTG GTA CCT TAC

Supplementary Table 4. Sequencing primers used for HMP013 PvDBP_RII.

References

1. Sridhar S, Reyes-Sandoval A, Draper SJ, Moore AC, Gilbert SC, Gao GP, Wilson JM, and Hill AV. Single-dose protection against Plasmodium berghei by a simian adenovirus vector using a human cytomegalovirus promoter containing intron A. *J Virol.* 2008;82(8):3822-33.
2. Douglas AD, de Cassan SC, Dicks MD, Gilbert SC, Hill AV, and Draper SJ. Tailoring subunit vaccine immunogenicity: Maximizing antibody and T cell responses by using combinations of adenovirus, poxvirus and protein-adjuvant vaccines against Plasmodium falciparum MSP1. *Vaccine.* 2010;28(44):7167-78.
3. Sheehy SH, Duncan CJ, Elias SC, Biswas S, Collins KA, O'Hara GA, Halstead FD, Ewer KJ, Mahungu T, Spencer AJ, et al. Phase Ia clinical evaluation of the safety and immunogenicity of the Plasmodium falciparum blood-stage antigen AMA1 in ChAd63 and MVA vaccine vectors. *PloS one.* 2012;7(2):e31208.
4. Douglas AD, Williams AR, Knuepfer E, Illingworth JJ, Furze JM, Crosnier C, Choudhary P, Bustamante LY, Zakutansky SE, Awuah DK, et al. Neutralization of Plasmodium falciparum Merozoites by Antibodies against PfRH5. *J Immunol.* 2014;192(1):245-58.
5. Hjerrild KA, Jin J, Wright KE, Brown RE, Marshall JM, Labbe GM, Silk SE, Cherry CJ, Clemmensen SB, Jorgensen T, et al. Production of full-length soluble Plasmodium falciparum RH5 protein vaccine using a Drosophila melanogaster Schneider 2 stable cell line system. *Sci Rep.* 2016;6(30357.
6. Quan J, and Tian J. Circular polymerase extension cloning of complex gene libraries and pathways. *PLoS One.* 2009;4(7):e6441.
7. Jin J, Hjerrild KA, Silk SE, Brown RE, Labbe GM, Marshall JM, Wright KE, Bezemer S, Clemmensen SB, Biswas S, et al. Accelerating the clinical development of protein-based vaccines for malaria by efficient purification using a four amino acid C-terminal 'C-tag'. *Int J Parasitol.* 2017.
8. Batchelor JD, Zahm JA, and Tolia NH. Dimerization of Plasmodium vivax DBP is induced upon receptor binding and drives recognition of DARC. *Nat Struct Mol Biol.* 2011;18(8):908-14.
9. Choe H, Moore MJ, Owens CM, Wright PL, Vasilieva N, Li W, Singh AP, Shakri R, Chitnis CE, and Farzan M. Sulphated tyrosines mediate association of chemokines and Plasmodium vivax Duffy binding protein with the Duffy antigen/receptor for chemokines (DARC). *Mol Microbiol.* 2005;55(5):1413-22.
10. Douglas AD, Baldeviano GC, Lucas CM, Lugo-Roman LA, Crosnier C, Bartholdson SJ, Diouf A, Miura K, Lambert LE, Ventocilla JA, et al. A PfRH5-Based Vaccine Is Efficacious against Heterologous Strain Blood-Stage Plasmodium falciparum Infection in Aotus Monkeys. *Cell host & microbe.* 2015;17(1):130-9.
11. de Cassan SC, Shakri AR, Llewellyn D, Elias SC, Cho JS, Goodman AL, Jin J, Douglas AD, Suwanarusk R, Nosten FH, et al. Preclinical Assessment of Viral Vectored and Protein Vaccines Targeting the Duffy-Binding Protein Region II of *Plasmodium Vivax*. *Front Immunol.* 2015;6(348.
12. McCarthy JS, Griffin PM, Sekuloski S, Bright AT, Rockett R, Looke D, Elliott S, Whiley D, Sloots T, Winzeler EA, et al. Experimentally induced blood-stage *Plasmodium vivax* infection in healthy volunteers. *The Journal of infectious diseases.* 2013;208(10):1688-94.
13. Zimin AV, Marcais G, Puiu D, Roberts M, Salzberg SL, and Yorke JA. The MaSuRCA genome assembler. *Bioinformatics.* 2013;29(21):2669-77.
14. Assefa S, Keane TM, Otto TD, Newbold C, and Berriman M. ABACAS: algorithm-based automatic contiguation of assembled sequences. *Bioinformatics.* 2009;25(15):1968-9.
15. Auburn S, Bohme U, Steinbiss S, Trimarsanto H, Hostetler J, Sanders M, Gao Q, Nosten F, Newbold CI, Berriman M, et al. A new Plasmodium vivax reference sequence with improved

assembly of the subtelomeres reveals an abundance of pir genes. *Wellcome open research.* 2016;1(4.

16. Steinbiss S, Silva-Franco F, Brunk B, Foth B, Hertz-Fowler C, Berriman M, and Otto TD. Companion: a web server for annotation and analysis of parasite genomes. *Nucleic Acids Res.* 2016;44(W1):W29-34.
17. Sheehy SH, Duncan CJ, Elias SC, Collins KA, Ewer KJ, Spencer AJ, Williams AR, Halstead FD, Moretz SE, Miura K, et al. Phase Ia Clinical Evaluation of the Plasmodium falciparum Blood-stage Antigen MSP1 in ChAd63 and MVA Vaccine Vectors. *Mol Ther.* 2011;19(12):2269-76.
18. Williams AR, Douglas AD, Miura K, Illingworth JJ, Choudhary P, Murungi LM, Furze JM, Diouf A, Miotto O, Crosnier C, et al. Enhancing blockade of Plasmodium falciparum erythrocyte invasion: assessing combinations of antibodies against PfRH5 and other merozoite antigens. *PLoS pathogens.* 2012;8(11):e1002991.
19. Hodgson SH, Choudhary P, Elias SC, Milne KH, Rampling TW, Biswas S, Poulton ID, Miura K, Douglas AD, Alanine DG, et al. Combining viral vectored and protein-in-adjuvant vaccines against the blood-stage malaria antigen AMA1: report on a phase 1a clinical trial. *Molecular therapy : the journal of the American Society of Gene Therapy.* 2014;22(12):2142-54.
20. Hostetler JB, Sharma S, Bartholdson SJ, Wright GJ, Fairhurst RM, and Rayner JC. A Library of Plasmodium vivax Recombinant Merozoite Proteins Reveals New Vaccine Candidates and Protein-Protein Interactions. *PLoS Negl Trop Dis.* 2015;9(12):e0004264.
21. Elias SC, Choudhary P, de Cassan SC, Biswas S, Collins KA, Halstead FD, Bliss CM, Ewer KJ, Hodgson SH, Duncan CJ, et al. Analysis of human B-cell responses following ChAd63-MVA MSP1 and AMA1 immunization and controlled malaria infection. *Immunology.* 2014;141(4):628-44.
22. Shakri AR, Rizvi MM, and Chitnis CE. Development of quantitative receptor-ligand binding assay for use as a tool to estimate immune responses against Plasmodium vivax Duffy binding protein Region II. *J Immunoassay Immunochem.* 2012;33(4):403-13.
